# Towards improved ecosystem service assessments in marine systems: A systematic review and evaluation of effectiveness

**DOI:** 10.1007/s13280-025-02299-2

**Published:** 2025-11-07

**Authors:** Juliana Socrate, Aurelija Armoskaite, Víctor Cordero Penín, Débora Gutierrez, Miriam von Thenen

**Affiliations:** 1 Coastal Sea Geography Group, Leibniz Institute for Baltic Sea Research Warnemünde, Rostock, Germany; 2 Latvian Institute of Aquatic Ecology, Riga, Latvia; 3 Biodiversity and Conservation Group (BIOCON), IU-ECOAQUA, University of Las Palmas de Gran Canaria, 35017 Canary Islands, Spain; 4 Faculty of Sciences and Technology (FCT), University of the Azores (UAc), Rua da Mãe de Deus, 9500-321 Ponta Delgada, Portugal; 5 Marine and Environmental Sciences Centre (MARE), Aquatic Research Network (ARNET), Faculty of Sciences, University of Lisbon, 1749-016 Lisbon, Portugal; 6 Faculty of Sciences and Technology (FCT), Universidade do Algarve, Gambelas Campus, 8005-139 Faro, Portugal; 7 Centre for Marine and Environmental Research (CIMA), Aquatic Research Network (ARNET), University of Algarve, Gambelas Campus, 8005-139 Faro, Portugal

**Keywords:** Ecosystem service assessments, Evaluation of effectiveness, Marine ecosystems, Regional comparisons, Sustainable marine planning

## Abstract

Ecosystem service assessments (ESA) are widely used to support marine management, yet their application varies across regions due to differences in governance frameworks, data availability, and methodological approaches. This study systematically reviews ESA methods and tools applied in offshore areas of the Baltic Sea, South Atlantic Ocean, and Western Mediterranean Sea, evaluating their effectiveness in supporting sustainable marine management with a specific focus on offshore wind farms, marine protected areas, and fishing activities. The findings reveal geographical variations in ESA implementation, highlighting strengths and limitations in their ability to inform policy and decision-making. While methodological diversity enhances ESA’s capacity to capture complex socio-ecological interactions, inconsistencies in application reduce its effectiveness. Addressing these gaps requires refining methodological approaches, strengthening interdisciplinary collaboration, and improving the link between ESA and marine governance. This research provides insights into the current state of ESA applications and offers recommendations for enhancing their role in ecosystem-based marine planning and policy integration.

**Supplementary Information:**

The online version contains supplementary material available at 10.1007/s13280-025-02299-2.

## Introduction

Ecosystem services assessments (ESA) play a critical role in environmental management by identifying and evaluating the benefits that ecosystems provide to society. Increasing human activities place growing pressure on marine environments, altering ecosystem functions and reducing their capacity to provide essential ecosystem services (ES) (Costanza et al. 1997; Steffen et al. 2015).

Assessing the capacity of ecosystems to supply specific services requires the use of diverse methods and tools. To effectively evaluate ecosystem condition, service provision, and the resulting implications for human well-being, integrated approaches are essential. These assessments enable decision-makers to identify and prioritize the most valued services, thereby supporting more sustainable management strategies (Alcamo et al. 2003). A wide array of methods has been employed in ESA, differing in levels of complexity, data needs, and spatial and temporal resolution. These methods range from quantitative techniques (such as biophysical modeling and economic valuation, which support standardized and replicable assessments) to qualitative methods like participatory mapping and expert-based evaluations, which capture social and perceptual dimensions of ES (de Groot et al. 2010; Liquete et al. 2013).

ESA has become particularly relevant in marine governance, supporting marine spatial planning (MSP), marine protected areas (MPAs), and fisheries management (Böhnke-Henrichs et al. 2013; Lester et al. 2013). By providing a structured approach to balancing conservation and resource use, ESA supports decision-making processes that integrate ecological, economic, and social considerations (Barbier 2017). Sustainable marine management depends on aligning conservation objectives with socioeconomic activities, requiring robust ESA methods and tools that can capture ecological functions, human dependencies, and respond to varying governance and socio-institutional contexts. MSP, in particular, could benefit from ESA by integrating spatial and temporal dynamics, which help mitigate conflicts among competing activities and support sustainable marine use. However, regional differences in policy frameworks, resource availability, and institutional capacities influence ESA applications, shaping how ESs are valued and managed (Sun et al. 2018).

Effectiveness in the context of ESA refers to their capacity to inform and support real-world decision-making through comprehensive, context-relevant, and implementable analyses. As emphasized by Kukkala and Moilanen (2013), effectiveness extends beyond efficiency: it must be assessed relative to specific goals and must reflect the adequacy, applicability, and long-term viability of solutions. In marine management, this includes the ability of ESAs to integrate ecological, social, and economic dimensions, address spatial and temporal trade-offs, and engage relevant stakeholders. Effectiveness is also shaped by enabling conditions such as human, social, and financial capital (Knight et al. 2011), influencing how assessment outcomes are translated into action.

ESA effectiveness is particularly relevant in the context of marine activities such as fisheries, offshore wind farms (OWFs), and MPAs, where management decisions depend on spatial prioritization, legitimacy, and policy relevance (Corrales et al. 2020). Enhancing ESA effectiveness requires balancing methodological consistency (essential for cross-case comparability) with the flexibility needed to reflect the socio-ecological specificities of each region and activity. Additionally, given the growing complexity of marine governance, refining ESA methods is essential to maintaining their relevance for decision-making under changing ecological and socioeconomic conditions (Koundouri et al. 2023).

The aim of this paper is to assess the effectiveness of different ESA methods and tools in supporting sustainable marine management. The following research questions were posed:

- What are the geographical differences and similarities in ESA applications, and how do they vary across activities?
- What are the main challenges, strengths, policy implications, and trade-offs identified in the ESA studies and the applied methods and tools?
- What is the effectiveness of different ESA methods and tools in supporting sustainable management?

To address these research questions, a systematic review was conducted to identify case studies with ESA methods and tools applied in the Baltic Sea (BS), South Atlantic Ocean (SAO), and Western Mediterranean Sea (WMS) that enabled the analysis of regional and sectoral patterns, followed by an evaluation of their ESA effectiveness. This paper builds on existing approaches to develop an analytical methodology for evaluating ESA, applying it as the main lens to assess how current ESA support MSP and marine management across diverse socio-ecological contexts. The methodology provides a structured and transferable approach that can synthesize diverse ESA methods and tools, and enable systematic comparison across cases. The analysis focuses on three key maritime activities (fishing, MPAs, and OWFs) that are central to current marine governance debates, MSP requirements, governance challenges and represent diverse ES demands by society (White et al. 2012; Inácio et al. 2024; Zaucha et al. 2025).

The methodology is detailed in "Materials and methods" section. "Geographical areas and activities under study" section describes the selected regions and activities, and the reasoning behind their choice. "Systematic literature review process" and "Data extraction and categorization" sections describe how case studies were selected, how information was extracted, and how data were analyzed to respond to the first two research questions. "Evaluation of ESA methods and tools" section outlines the evaluative methodology developed to assess ESA effectiveness across studies. Results are presented in "Results" section, where each research question is addressed in a dedicated subsection. Finally, "Discussion" section provides a critical discussion of the main findings, highlighting their implications for improving ESA methods and tools, and their integration into marine management and planning processes.

## Materials and methods

### Geographical areas and activities under study

This study examines the application of ESA methods and tools in three contrasting marine regions: the BS, WMS, and SAO.

The selected regions reflect highly diverse socio-ecological systems and governance frameworks, offering a basis for comparing ESA applications under varying conditions of data availability, institutional capacity, and sectoral development. The rationale for selecting these areas is not solely based on data richness, but also on their differing policy contexts, ecological pressures, and levels of ESA integration. Importantly, the study focuses on offshore areas (up to 200 nautical miles), thereby excluding coastal studies to ensure a consistent analytical scope and enhance comparability across regions.

A summary of ecological, institutional, and policy differences among regions is provided in Table 1, which outlines key characteristics from the different geographical region. The elements summarized were selected based on recurrent factors identified in the ESA and marine governance literature as key drivers of assessment design and implementation. These contrasts support the selection of case study areas and frame the comparative analysis of ESA practices aimed at supporting sustainable marine management across diverse governance and socio-ecological contexts.

**Table 1:** Key characteristics of the BS, WMS, and SAO relevant to the study. The table summarizes regional differences in governance structures, data availability, management challenges, policy relevance, and presence of target activities (fisheries, MPAs, OWF). These contrasts illustrate the socio-ecological diversity of the selected areas and support their inclusion as case study regions to explore ESA methods variation and effectiveness. Data compiled from UNESCO-IOC (2021) for the WMS, CEPAL (2025) for SAO, and HELCOM (2025) for the BS. Additional data points applicable to all three regions were obtained from the World Bank Group (2025)

Element	Baltic Sea (BS)	Western Mediterranean Sea (WMS)	South Atlantic Ocean (SAO)
Institutional integration	Regional coordination (e.g., HELCOM)	Fragmented governance, multiple overlapping jurisdictions	Low integration, governance varies widely across countries
Data availability	High—long-term monitoring programs, transboundary datasets	Moderate—uneven data by country	Low—data gaps in offshore systems, especially for ES
Dominant management challenges	Eutrophication, overfishing, spatial conflicts (e.g., OWFs vs MPAs)	High biodiversity pressure, tourism, fishing conflicts	Emerging marine uses, fisheries overexploitation, lack of MPA networks
Development of ESA practices	Advanced—ES integrated into some policy tools	Moderate—growing interest, still limited integration in practice	Emerging—ESA mostly academic or exploratory
Presence of target activities	All three: well-established fisheries, MPAs, and expanding OWFs	All three: MPAs widespread, coastal fishing, some OWFs	Fisheries dominant; MPAs limited; OWFs not yet operational, but projected
Relevance for MSP	High—region actively engages in marine spatial planning	Growing—EU MSP Directive in implementation	Emerging—MSP under development in some countries
Socioeconomic diversity	Mostly EU countries with coordinated policies	EU countries, varied development levels	High—diverse socioeconomic and institutional contexts

Fisheries represent long-established, extractive uses and are often among the most socioeconomically important activities for coastal communities (Inácio et al. 2024). They directly depend on fish stocks and numerous ecosystem functions (e.g., healthy spawning and nursery habitats, feeding grounds, and refuge and shelter) sustaining these stocks (Inácio et al. 2024). Wild-caught fish provide food and other resources, may carry cultural heritage value, and contribute to regulating services such as pest and disease regulation (Murphy et al. 2021). However, exploitation has put many stocks at risk of collapse, prompting management efforts to promote more sustainable fisheries (Murphy et al. 2021).

MPAs reflect conservation-oriented governance approaches (Grorud-Colvert et al. 2021). Areas for protection are designated to maintain a well-functioning ecosystem and the supply of ES, e.g., providing carbon sequestration, spawning and nursery habitats for other species, an environment for recreation and tourism (Inácio et al. 2024) and achieve increasing biodiversity protection goals, e.g., the Kunming-Montreal Global Biodiversity Framework (Convention on Biological Diversity 2022). MPAs restrict some activities and access to certain in-demand ES to protect habitats from pressures (Grorud-Colvert et al. 2021). However, the ecological benefits, such as spillover of juvenile and adult fish, support the recovery and the supply of others, e.g., fish for food (Pita et al. 2024).

OWFs symbolize emerging, spatially demanding developments, often seen steering the marine planning and management process and a dialogue for multi-use (White et al. 2012). Although OWFs are not currently operational in the SAO, this region was retained to evaluate ESA practices in existing maritime sectors and to assess preparedness for future offshore energy expansion, as projected by the World Bank Group (2025).

### Systematic literature review process

A systematic review framework guided by the Preferred Reporting Items for Systematic Reviews (PRISMA) protocol (Haddaway et al. 2022) was employed to ensure a rigorous and transparent synthesis of existing knowledge. The aim of the systematic review was to identify, classify, and critically analyze case studies applying ESA across the three selected marine regions (BS, WMS, and SAO) focusing on three relevant activities: fisheries, MPAs, and OWFs. This review was designed to address the overarching objective of the study: to assess the effectiveness of ESA in supporting sustainable marine management across diverse socio-ecological contexts.

To streamline the review process and increase consistency during article screening and selection, the PICO Portal software was used, integrating artificial intelligence (AI) and machine learning functionalities. The AI component facilitated the identification of key concepts and terminology aligned with the project’s predefined inclusion and exclusion criteria by highlighting corresponding words in the title, abstract, and keywords, which speed up the screening process. The AI also supported the data extraction phase by highlighting relevant sections of the text and organizing them for review (for details, see "Data extraction and categorization" section). A summary of the key elements necessary for a systematic review according to PRISMA standards is in the Supplementary Information (SI)—Annex A.

#### Literature search strategy

Searches were conducted across Web of Science, Scopus, and SciELO. The search strings targeted ESA methods and tools, ESs, and the geographical regions and activities described above. Articles in English, Spanish, and Portuguese were included to ensure comprehensive regional representation, based on the authors’ experience and confirmed by regional databases (e.g., SciELO). In this latter source, relevant studies from SAO are often written in Spanish or Portuguese. Conversely, European marine research is usually published in English, particularly in the context of EU-funded projects and academic institutions. The specific search string and the number of articles per browser can be found in SI—Annex B. From an initial pool of 4078 articles (196 from SciELO, 300 from Scopus, and 3582 from Web of Science), a deduplication process using PICO Portal (2024) resulted in 3925 unique articles.

#### Screening and selection process

**Fig. 1:**
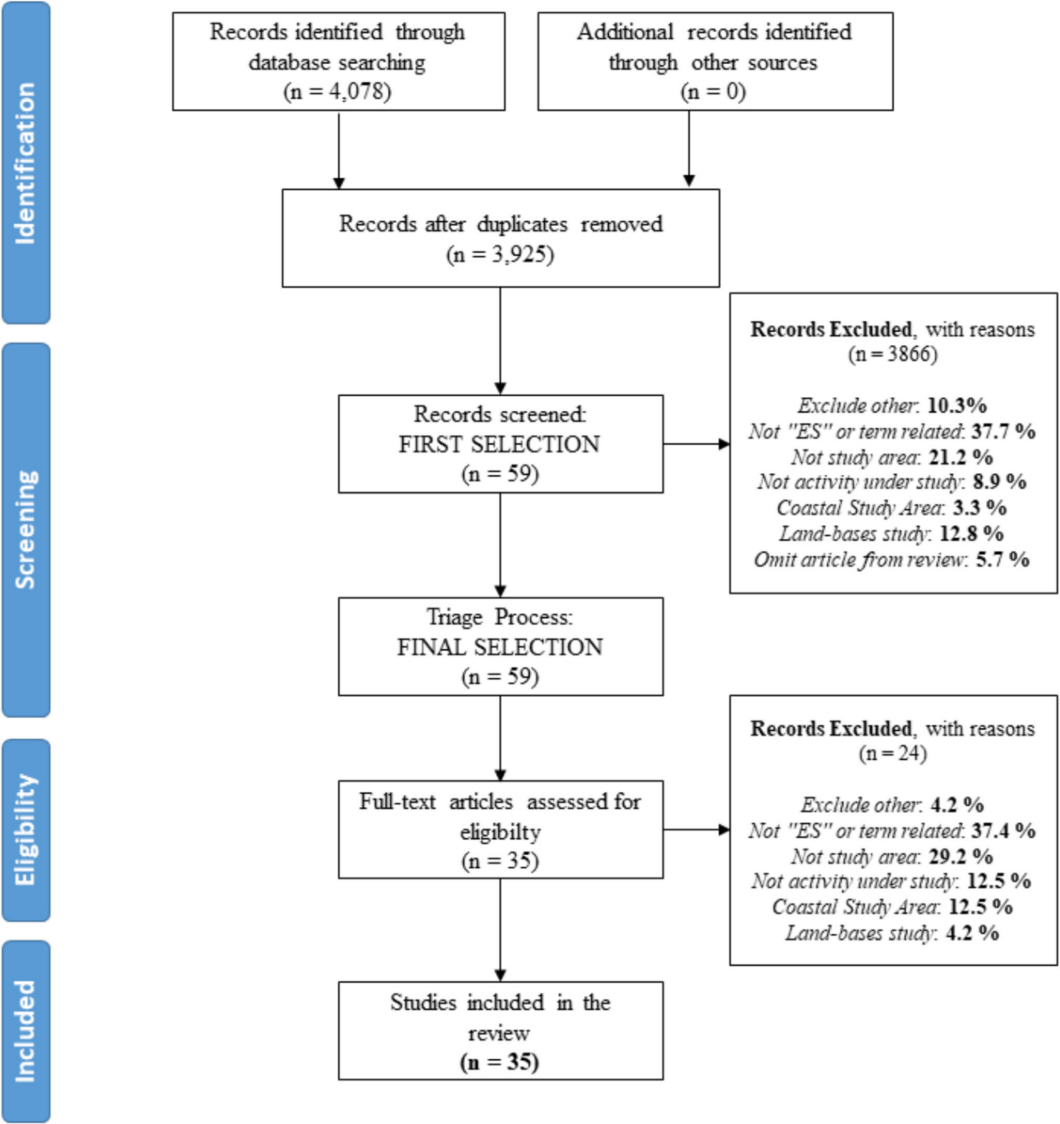
Flow diagram of the methodology and selection processes used in this systematic review. It follows the rules and templates of PRISMA (Haddaway et al. 2022)

Using PICO Portal’s AI-enhanced tools, articles were screened in two stages: abstract review and full-text review. During the first stage, dual reviewer agreement was required for inclusion, with a third reviewer resolving conflicts. Exclusion criteria included the absence of ES references, lack of focus on the specified geographical areas or activities, and studies focused solely on terrestrial ecosystems. A PRISMA flow diagram (Fig. 1) summarizes the selection process. Following screening, 59 articles were shortlisted, and 35 case studies were selected for final data extraction (SI—Annex C).

### Data extraction and categorization

#### Extraction process

The data extraction process encompassed 23 categories, detailed in SI—Annex D. PICO Portal facilitated this process by structuring extraction around categories predefined by the author team, including general information (e.g., title, publication year, study aim); geographical region (BS, WMS, SAO); activity (fishing, MPAs, OWFs); ESA methods and tools; ES analyzed; and key analytical aspects such as trade-offs, policy implications, and challenges.

To enhance accuracy and consistency, each extraction category was paired with a set of keywords, proxies, and guiding questions, which were input into the AI-assisted interface of the software. These elements helped train the software to recognize relevant content in the full texts and highlight sections where potential answers were located. After this, the AI would automatically suggest responses to each category based on its detection of relevant textual cues. These suggestions were compiled into an exportable Excel file, which facilitated structured data handling.

Validation was conducted through a double-blind review of 20 papers, which revealed inconsistencies in the answers of six categories. These issues were addressed by refining the keyword and proxy criteria and manually re-evaluating the affected entries to ensure reliability across the dataset.

#### Categorization of ESA information

The 23 categories extracted through PICO Portal included both open-ended responses and predefined multiple-choice selections. Open-ended responses (e.g., main aim of the paper) were categorized thematically, while structured fields (e.g., geographical region or activity) followed predefined classification options (e.g., BS, SAO, WMS). The objective of this categorization was twofold: to provide a systematic and comprehensive characterization of the selected case studies and to support the analytical framework needed to answer the first two research questions (related to regional and sectoral differences) and their relation to ESA challenges, strengths, policy implications, and trade-offs.

Themes for open-ended responses were derived through an inductive process, based on the content extracted from each study. For each category, the research team reviewed all responses suggested by the AI and clustered them according to semantic similarity and analytical relevance. For instance, for the "study aim" field, two levels of classification were developed: one based on methodological orientation (e.g., ES assessment, ecosystem-based management, etc.), and another capturing the thematic focus (e.g., MSP, biodiversity conservation, or socio-ecological interactions, among others). This approach ensured internal consistency and transparency, enabling future replicability. The categorization criteria and group definitions were established iteratively and recorded in detail in SI—Annex E—Definitions.

The resulting categorization was not only essential for describing the sample of reviewed studies, but also served as a foundation for evaluating ESA effectiveness ("Evaluation of ESA methods and tools" section), by highlighting contextual and methodological focus, relevant to interpreting performance. The outputs of this classification process are fully presented in SI—Annex E—Analysis.

#### Data analysis

The data analysis aimed to synthesize and compare patterns emerging from the selected case studies across geographical regions and sectors, serving as the empirical basis for subsequent evaluation of ESA methods and tools. This phase built upon the outputs of the extraction and categorization process (see "Extraction process" and "Categorization of ESA information" sections), enabling a structured characterization of the evidence base in relation to the first and second research questions.

Analyses were conducted using RStudio. The dataset was filtered by geographical region and by activity to identify and interpret contextual differences in ESA approaches. Descriptive statistics and frequency analyses were used to summarize the data.

This analytical approach contributed directly to answering Research Questions 1 and 2, allowing for a nuanced characterization of the reviewed cases and laying the groundwork for evaluating ESA effectiveness in diverse governance and ecological contexts.

### Evaluation of ESA methods and tools

#### A methodology for evaluating effectiveness

In this study, effectiveness refers to their capacity to inform and support real-world decision-making through comprehensive, context-relevant, and implementable analyses. The definition and the methodology for structuring the evaluation of tools and methods draw from established literature examining the links between ESA and decision-making processes, namely Everard and Waters (2013), Ervin et al. (2014) and Gee et al. (2019).

Ervin et al. (2014) developed ten principles to guide assessments of ES values arising from the need to assess the social, ecological, and economic benefits of ecosystems and biodiversity. Their principles aimed to ensure comprehensive, credible, and consistent assessments capable of informing decision-making and promote long-term sustainability.

Everard and Waters (2013) highlighted the role of ESA as a decision-support tool, capable of identifying unintended consequences and optimize benefits. The level of detail required in an assessment was context-dependent, and the application of ESA facilitated risk assessment, impact evaluations, and stakeholder engagement. Their work highlighted the necessity of integrating ES assessments into broader policy and management frameworks.

Gee et al. (2019) proposed a problem–question-based structure for tool evaluation in the context of MSP. Their methodology assessed the potential benefits of tool use against four common integration challenges in MSP (multi-level and transboundary, policy and sector, stakeholder, and knowledge integration). Specific endpoints were defined for each challenge, including general desired outcomes of integrated MSP processes, to serve as a template for assessment.

While Ervin et al. (2014) provided a normative set of principles, Everard and Waters (2013) highlight the functional and contextual considerations for effective ESA, and Gee et al. (2019) introduced a diagnostic, question-led approach. Combining these perspectives enables a methodological evaluation that safeguards that assessments are systematically structured while remaining adaptable to diverse contexts.

#### Adapting the methodology to the ESA context

The Ervin et al. (2014) guiding principles were used as a foundation for the evaluation of ESA effectiveness, but required adaptation, as their original focus was on the evaluation of ES. Building on the lessons learned from Everard and Waters (2013), these principles were refined. For instance, Ervin et al. (2014) stated in their guiding principle 3: *Identify and engage all interested and affected stakeholders in a transparent, inclusive manner.* Similarly, Everard and Waters (2013) argue that *a systems approach recognizes all stakeholders in decision-making, as all ecosystem services represent the interests and value systems of different sectors of society.* The convergence of these perspectives reinforced the inclusion of *Stakeholder Engagement* as one of the effectiveness principles, highlighting the need for representation from all relevant actors and for transparent, inclusive decision-making processes.

This refinement process resulted in a final set of ten effectiveness principles: *Scalability and Flexibility, Integration of Multiple Dimensions, Policy Relevance, Stakeholder Engagement, Comprehensive Scope, Scientific Rigor, Transparency and Reproducibility, Practical Feasibility, Dynamic and Adaptive Approach,* and* Interdisciplinary Approach.*

Similarly, the five main challenges identified by Gee et al. (2019) for MSP tool evaluation were adjusted to apply more broadly to ESA, and to align with the ten effectiveness principles. Each challenge was reviewed against the principles, retaining four of Gee et al.’s original challenges and adding two new ones to ensure comprehensive coverage. This process produced six key problems: *Problem 1: Multi-Scale and Multi-Dimensional Integration; Problem 2: Policy Relevance and Governance Support; Problem 3: Stakeholder Engagement and Inclusivity; Problem 4: Knowledge Integration and Scientific Robustness; Problem 5: Practical Feasibility and Adaptability; Problem 6: Comprehensive Scope and Ecosystem Approach.*

Following the logic of Gee et al. (2019), each key problem was specified through a set of evaluation questions, designed to translate broad challenges into operational criteria. For example, Problem 1: Multi-Scale and Multi-Dimensional Integration was linked to: 1.1. Does the method/tool address multiple geopolitical and/or geographical scales in its analysis? 1.2. Is the method/tool adaptable to different scales (local, regional, global)? 1.3. Does it integrate ecological, social, and economic dimensions effectively?

**Fig. 2:**
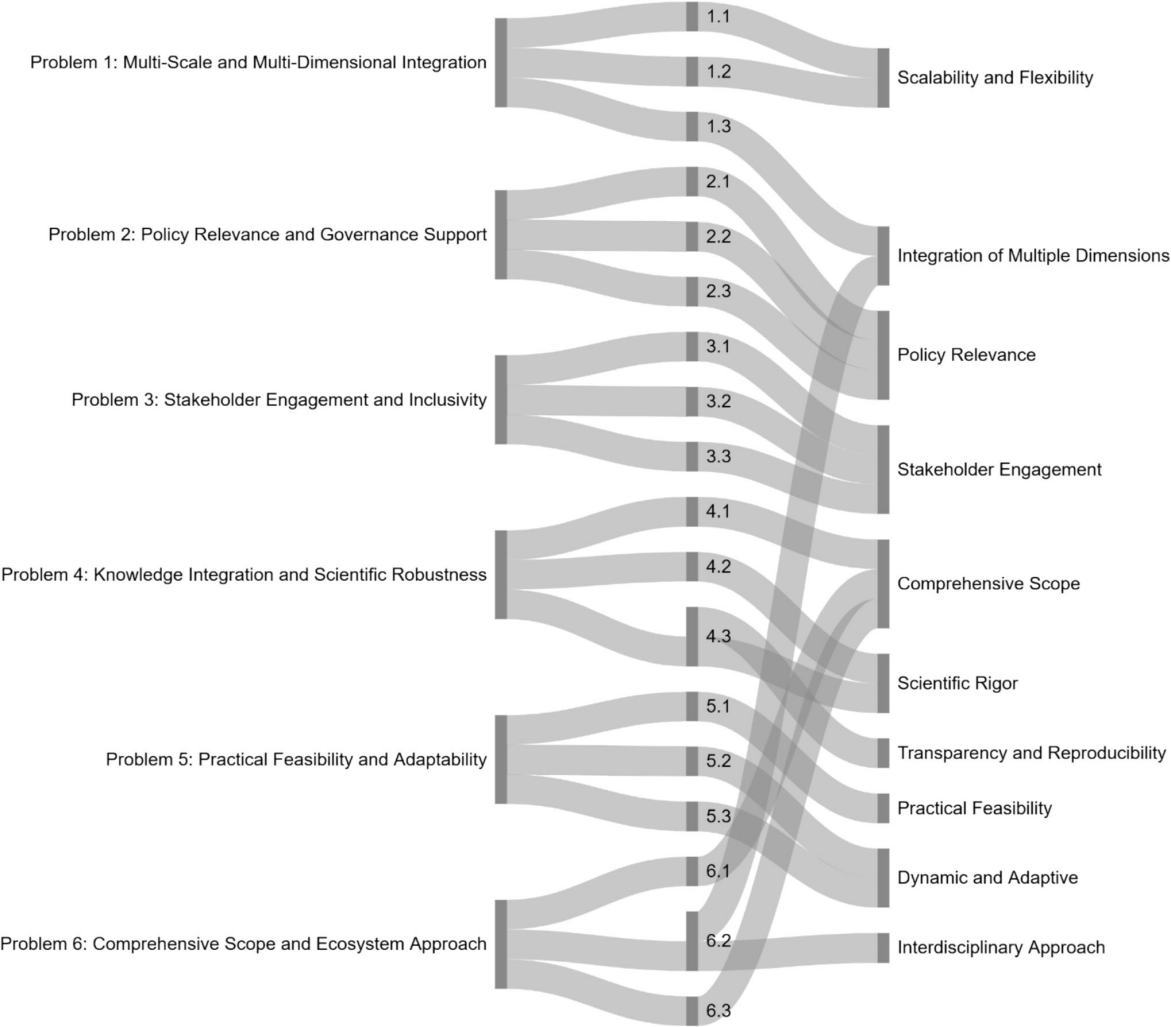
Relationships between key problems (left side of the diagram), specific evaluation questions (middle section; full list is available in the online resource (SI—Annex G), and guiding principles for assessing the effectiveness of ESA methods and tools (right side of the diagram)

Each problem was then linked to its evaluation questions, which in turn were associated with specific effectiveness principles. This structure is summarized in the Sankey diagram (Fig. 2), which depicts the relationships between key problems (left), evaluation questions (center; full list in SI—Annex G), and guiding principles (right).

The resulting methodology (expanded in SI—Annex G) was designed to enable systematic evaluation of ESA methods and tools against the defined effectiveness criteria. This approach not only facilitated the identification of strengths and weaknesses in ESA applications, but also provided a transparent basis for recommending targeted improvements.

#### Ranking of ESA methods and tools

To compare and rank the ESA methods and tools identified through the systematic literature review, the evaluation-question methodology was applied, with scores assigned accordingly (Fully Accomplished—2 points; Partially Accomplished—1 point; Not Accomplished—0 points). To avoid bias, the evaluation was conducted using the case study number, without identifying the authors of each study. The total scores were used to rank methods and tools, providing a comparative analysis of their performance (see SI—Annex F).

## Results

This results section is structured to address the three research questions. To answer the first two questions, the results of the systematic review are described in "What are the geographical differences and similarities in ESA applications, and how do they vary across activities?" and "What are the main challenges, strengths, policy implications, and trade-offs identified in the ESA studies and the applied methods and tools?" sections. For the third question, "What is the effectiveness of different ESA methods and tools in supporting sustainable management?" section presented the findings of 35 case study analyses following the earlier established evaluation methodology.

### What are the geographical differences and similarities in ESA applications, and how do they vary across activities?

#### Geographical-specific insights

A geospatial analysis of the reviewed studies revealed distinct trends in ESA applications, highlighting both concentrations and gaps in research coverage. Most case studies were located in the BS (17). The WMS followed with 10 studies, and the SAO with 9.

**Fig. 3:**
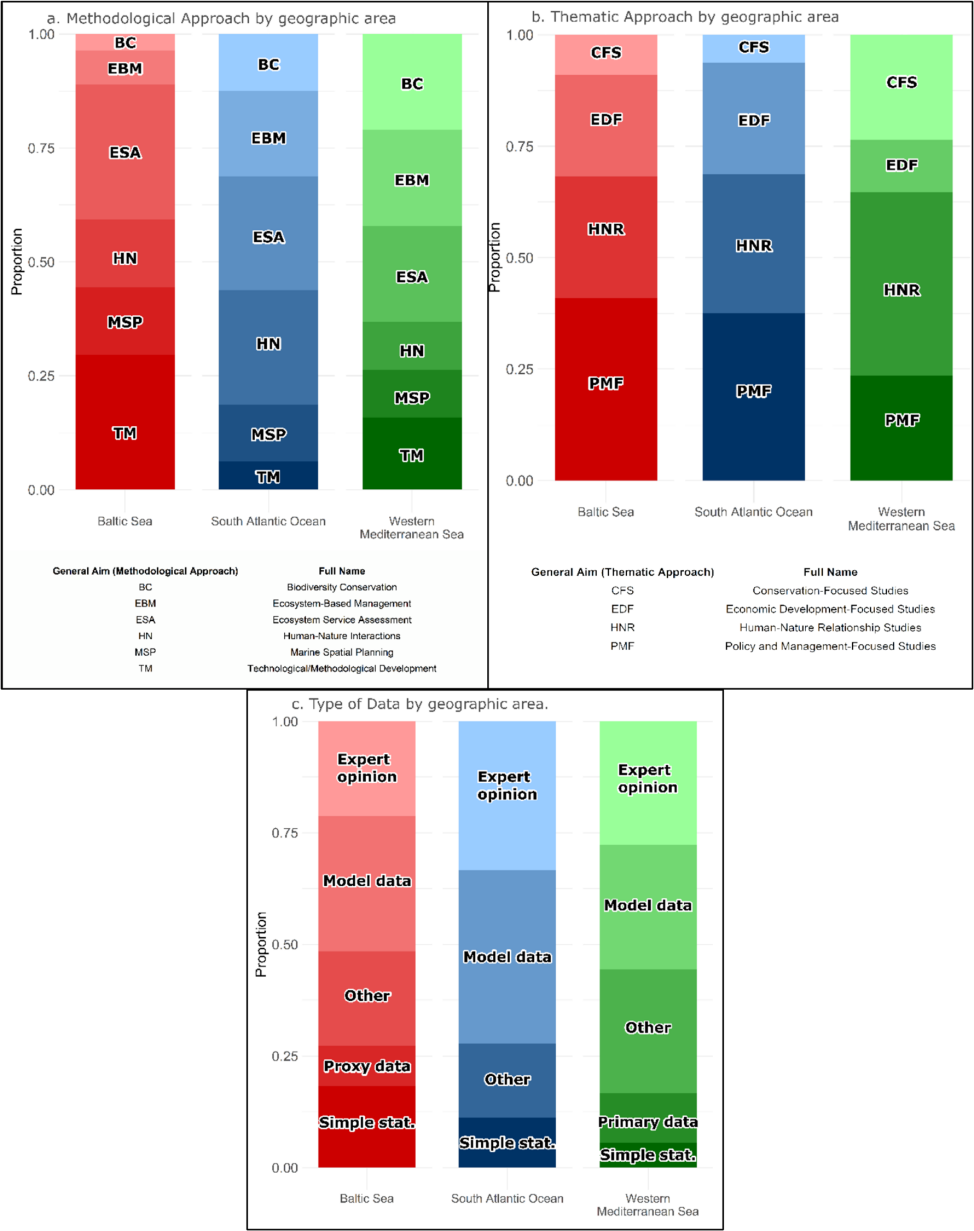
**a** Methodological Approach by geographical area. **b** Thematic Approach by geographical area. **c** Type of Data by geographical area. The category *Other* includes historical data, secondary data (from other studies), gray literature, official data from government institutions, and socioeconomic data from national sources. The abbreviation *Simple Stat* refers to *Simple Statistical Processes*

The analysis of the general aims of the reviewed studies indicates that *ESA* and *Human–Nature Interactions* were the most commonly applied methodological approaches across all three geographical zones (Fig. 3a). *ESA* was the most frequently used, particularly in the BS, where it was applied in 15 studies (e.g., Firth et al. 2016). *Human–Nature Interactions* approaches were also widely present, especially in the BS and SAO, where studies often explored socioeconomic drivers of fishery resource use, distinguishing between ES such as food provision and recreation (Brun et al. 2024). *Technological and Methodological Development* appeared primarily in studies from the BS and the WMS, with fewer instances in the SAO. *Marine Spatial Planning* approaches were relatively distributed across areas. In contrast, *Ecosystem-Based Management* was the least represented overall, although it had a more prominent role in the SAO, particularly in studies addressing the integration of ecological and socioeconomic components.

Thematic orientations of the reviewed studies (Fig. 3b) were primarily focused on *policy and management goals*, regardless of the methodological approach. This trend was particularly notable in the SAO (e.g., Ellif and Kikuchi 2017). *Human–Nature Relationship* oriented studies were most frequent in the WMS, while *Conservation-Focused* studies were concentrated in the same area but were comparatively scarce in the SAO. These studies often addressed MPAs or explored linkages between fisheries and biodiversity conservation goals. *Economic Development* oriented studies appeared across all geographical zones with less regional variation.

The analysis of methodological design, data types, and spatial–temporal scales showed distinct geographical patterns among the reviewed case studies. A mixed-methods design was the most frequently applied (22 studies), combining qualitative and quantitative methods. Qualitative methods alone were used in 7 studies, focusing primarily on stakeholder engagement and policy evaluation; these were distributed across the three geographical zones (3 in the WMS, 2 in the BS, and 2 in the SAO). Purely quantitative methods, such as statistical modeling or numerical assessments, were less frequently employed, appearing in only 6 studies (e.g., Ressurreição et al. 2012).

Model data were the most used (24 studies), crucial for simulating ecosystem processes when empirical data is scarce. Expert opinions (19 studies) complemented modeling by addressing data gaps, while literature-based data and systematic reviews (18 studies) provided further support. Primary data collection (two studies) and proxy data (three studies) were rare (Fig. 3c).

Methodological designs varied across geography regions. In the BS studies, model data, often at supranational and national scales, integrated expert input for broad-scale management (e.g., Bastardie and Brown 2020). The WMS studies combined model data with literature reviews and expert opinions (Scemama et al. 2020), while the SAO studies relied more on expert opinions and secondary data (Brun et al. 2024).

Research was evenly distributed between local (21 studies) and supranational (20 studies) levels, with national (18 studies) and subnational (12 studies) assessments also contributing. Short- and medium-term scenarios were predominant, while long-term projections were less frequent, with fewer studies applying extended temporal horizons.

**Fig. 4:**
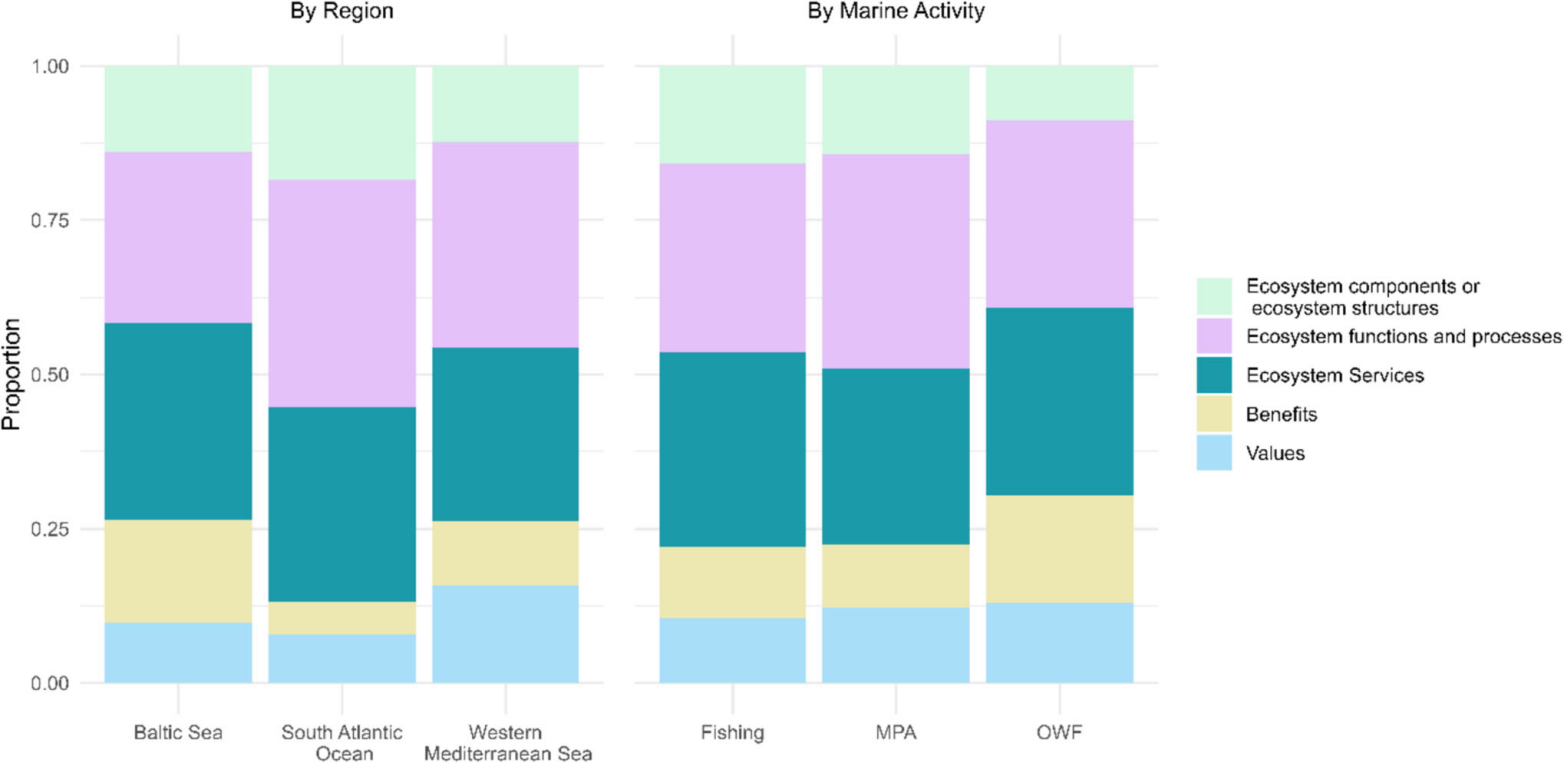
Distribution of the components of the ecosystem services cascade framework across geographical regions and human activities human activity: fishing, marine protected areas (*MPA*) and offshore wind farms (*OWF*). The components include ecosystem structures, functions, services, benefits, and values

To further understand how studies structured their analyses of ES, the application of the ES cascade framework was examined across the geographical areas. The framework includes five components: (1) ecosystem structures, (2) functions, (3) services, (4) benefits, and (5) values (Fig. 4). Among these, ecosystem functions and ecosystem services were the most commonly analyzed elements (e.g., Thrush and Dayton 2010), emphasizing the link between ecological processes and human well-being.

**Fig. 5:**
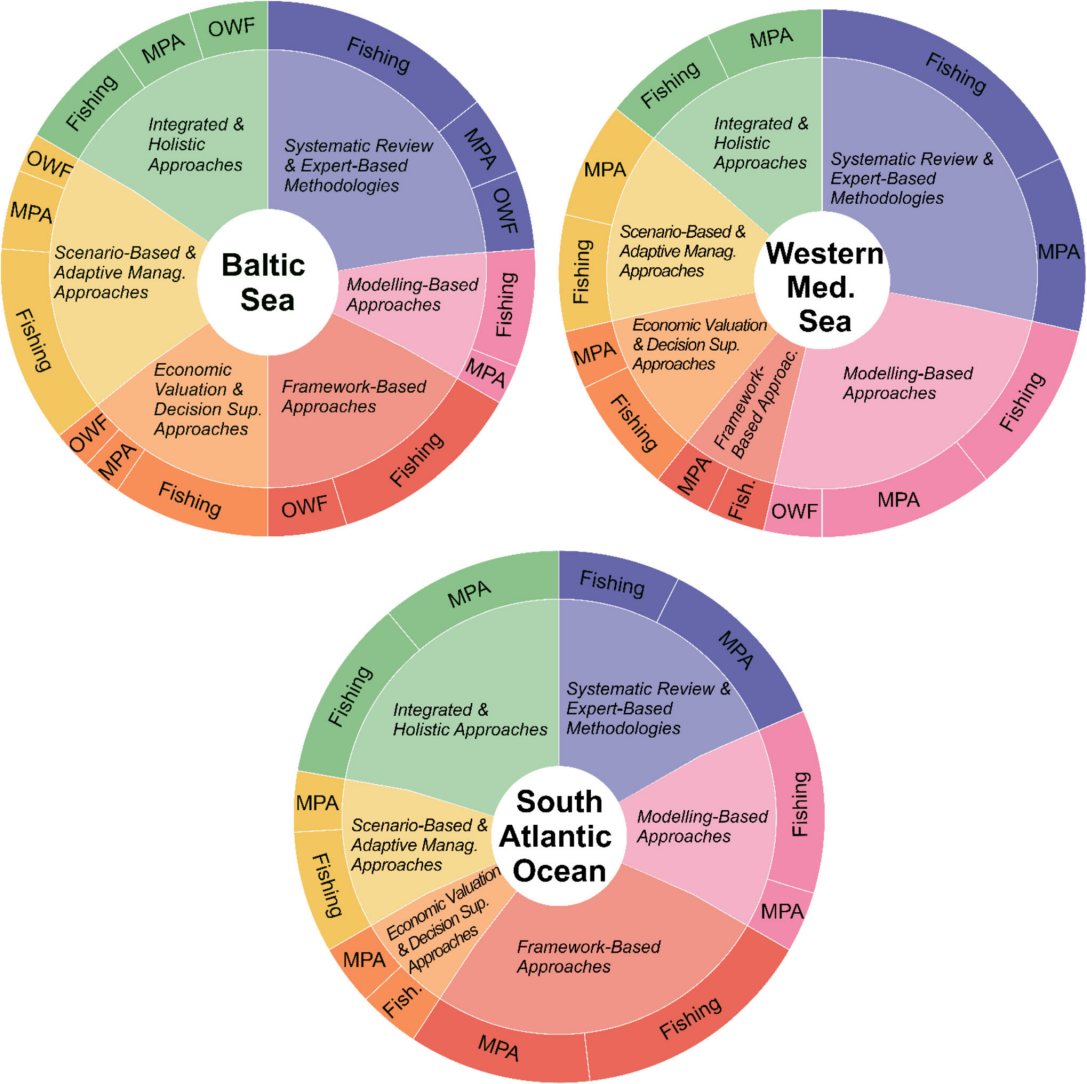
Sunburst chart showing the distribution of ESA methods across the three study areas: BS, WMS, and SAO. The inner ring represents the proportion of each ESA method used in each zone, while the outer ring illustrates the distribution of those methods by associated human activity: fishing (*Fish*.), offshore wind farms (*OWF*), and marine protected areas (*MPA*). ESA methods are *Systematic Review and Expert-Based Methodologies*; *Modeling-Based Approaches; Framework-Based Approaches; Economic Valuation and Decision-Support Approaches; Scenario-Based and Adaptive Management Approaches; Integrated and Holistic Approaches*

The distribution of methods across areas is shown in Fig. 5. The BS studies relied on *expert-based methods*, often complementing *modeling or scenario-based assessments* (Oinonen et al. 2016). The WMS studies exhibited strong use of *systematic reviews and expert-based methods*, combined with modeling techniques (Picone et al. 2017). *Framework-based approaches* were least prominent in the WMS, unlike the BS (Möllmann and Diekmann 2012) and SAO studies, where the Drivers-Activity-Pressures-State of change—Impacts and Responses (DAPSIR) was the most used framework (or its variant, DAPSIWR; Socrate et al. 2024).

With respect to tools, the analysis revealed that most studies relied solely on methods, with tools being utilized in fewer than 20% of cases. Among the tools applied, Ecopath with Ecosim (EwE) emerged as the most widely used across all three areas (e.g., Corrales et al. 2020). The BS exhibited the greatest variability in tool application, as evidenced by the presence of multiple distinct tools compared to the WMS and SAO (see SI—Annex E for more information).

#### Activity-specific insights

The analysis of ESA applications across different marine activities revealed distinct patterns, with some studies assessing multiple activities. Among the reviewed case studies, fishing was the most frequently analyzed activity (33 studies), followed by MPAs with 18 studies, and OWFs with 5. Geographically, the BS accounted for 15 fishing studies, 4 on OWFs, and 3 on MPAs. The WMS had 10 studies on fishing, 1 on OWFs, and 8 on MPAs. The SAO presented the lowest number of cases, with 8 focusing on fishing and 7 on MPAs. No studies on OWFs were recorded in the SAO, reflecting the absence of this activity in the area.

Regarding the application of the ES cascade framework across marine activities (Fig. 4), ecosystem functions and ecosystem services were the most frequently assessed in fishing case studies, reflecting their prevalence in the analyzed literature (e.g., Karydis 2023). In contrast, the value of ecosystem services was less frequently addressed in MPA-related studies (Le Cornu et al. 2014), while studies focused on fisheries and OWFs more commonly included later components of the cascade, such as benefits and values (Gasalla et al. 2004). MPA case studies more often considered the initial components (structures, functions, and services) with fewer references to benefits or values (Corrales et al. 2020).

When examining the relationship between ESA methods and marine activities (Fig. 5), *expert-based methods and systematic reviews* emerged as prominent across fisheries, MPAs, and OWFs. In the case of OWFs, the limited number of studies was associated with a narrower methodological representation. For MPAs case studies, *integrated and holistic approaches* were more frequently applied (e.g., Flávio et al. 2023), and appeared more often in relation to ecosystem assessments. *Framework-based approaches* were also present in studies addressing both fishing and OWF activities, supporting structured assessments of trade-offs and management strategies (e.g., Vieira Paiva et al. 2023).

As far as tools are concerned, their use was not observed in OWF studies, indicating a limited presence of technological applications reported in this sector for the sample analyzed. Conversely, the fisheries case studies exhibited considerable diversity in tool use, consistent with the higher number of studies addressing this activity (Ellif and Kikuchi 2017). The MPA studies demonstrated a more selective use of tools, with a primary focus on assessing ecosystem conditions rather than supporting economic decision-making (e.g., Maldonado et al. 2022).

### What are the main challenges, strengths, policy implications, and trade-offs identified in the ESA studies and the applied methods and tools?

#### Geographical-specific differences

The analysis of challenges, strengths, policy implications, and trade-offs revealed geographical-specific patterns across the case studies. The most frequently reported challenge was related to *data limitations, particularly data gaps and reliability* (e.g., Corrales et al. 2020), followed by the scarcity of *experts and potential bias* (Bryhn et al. 2020). In contrast, *economic vs. sustainability tensions* were less commonly noted (Picone et al. 2017).

Commonly cited strengths included *Policy Relevance and Management Insights* (Le Cornu et al. 2014), *Socio-Ecological Integration and Ecosystem-Based Approaches* (Firth et al. 2016). The WMS case studies focused on holistic planning and conflict resolution (Maldonado et al. 2022), while the SAO papers highlighted the importance of scalable and replicable methods (Topor et al. 2019).

Policy implications most often emphasized *Adaptive and Integrated Management Approaches*, including context-specific strategies for climate resilience and ecological shifts (e.g., Karydis 2023). *Conflict Resolution and International Cooperation* were less frequently addressed (e.g., Oinonen et al. 2016).

Trade-offs were dominated by tensions between *Economic Development and Conservation* (Hammer et al. 2003), with geographical differences such as *human well-being vs. exploitation* in the SAO case studies (Brun et al. 2024).

#### Activity-specific differences

Challenges in ESA applications varied across marine activities within the scope of the reviewed case studies, highlighting both shared and activity-specific obstacles. In fisheries-related studies (being the most represented in the sample), the most frequently reported challenges included *Problems with Data*, particularly issues related to data gaps, collection methods, and reliability. These were followed by *Limited Number of Experts and Potential for Expert Bias*, as well as *Uncertainty and Simplification in Modeling* and *Long-Term Projections* (e.g., Maldonado et al. 2022). *Economic Interests vs. Sustainability Goals* was the least frequently cited challenge in this group.

MPA-focused studies reflected similar patterns, with additional emphasis on *Knowledge Gaps and Challenges in the Applicability of ESA*, especially in contexts involving complex governance structures or the implementation of emerging conservation tools (e.g., Thrush and Dayton 2010). In contrast, OWF-related studies (fewer in number) reported a narrower range of challenges, primarily associated with *Multi-Scale Integration* and *Data Gaps*.

Strengths also varied by activity, with the fishing studies most frequently highlighting *Policy Relevance and Management Insights*, *Spatial Analysis and Scenario Modeling*, and *Holistic Approach and Conflict Resolution*, supported by data foundation (e.g., Sundblad et al. 2020). MPAs case studies placed greater emphasis on *Socio-Ecological Integration and the Ecosystem-Based Approach* (e.g., Le Cornu et al. 2014). *Stakeholder Inclusion* appeared least frequently across all activities, and was absent in OWF studies.

Policy implications showed consistent patterns across activities. In the fishing studies, the most frequently cited implication was the need for *Adaptive and Integrated Management Approaches*, which included multisectoral collaboration (e.g., Möllmann and Diekmann 2012), integration of cumulative impacts in planning (e.g., Muñoz et al. 2018), and linking ecological and socioeconomic dimensions to support policy uptake (e.g., Armoskaitė et al. 2020). This was followed by calls for *Stakeholder Engagement and Socioeconomic Integration*, particularly through participatory methods and expert validation (e.g., Uusitalo et al. 2023). The MPA studies followed a similar trajectory, with added emphasis on *Conservation and Climate Change Mitigation* (e.g., Thrush and Dayton 2010). The OWF studies shared many of these elements but lacked references to international cooperation or regulatory tools.

Trade-offs were most pronounced in the fishing studies, dominated by tensions between *Economic Development vs. Conservation* and *Balancing Human and Ecosystem Needs* (Vieira Paiva et al. 2023). The MPAs papers underscored *Conflicts Between Competing Activities*, especially within spatial planning.

#### Challenges and strengths of the ESA methods and tools

**Fig. 6:**
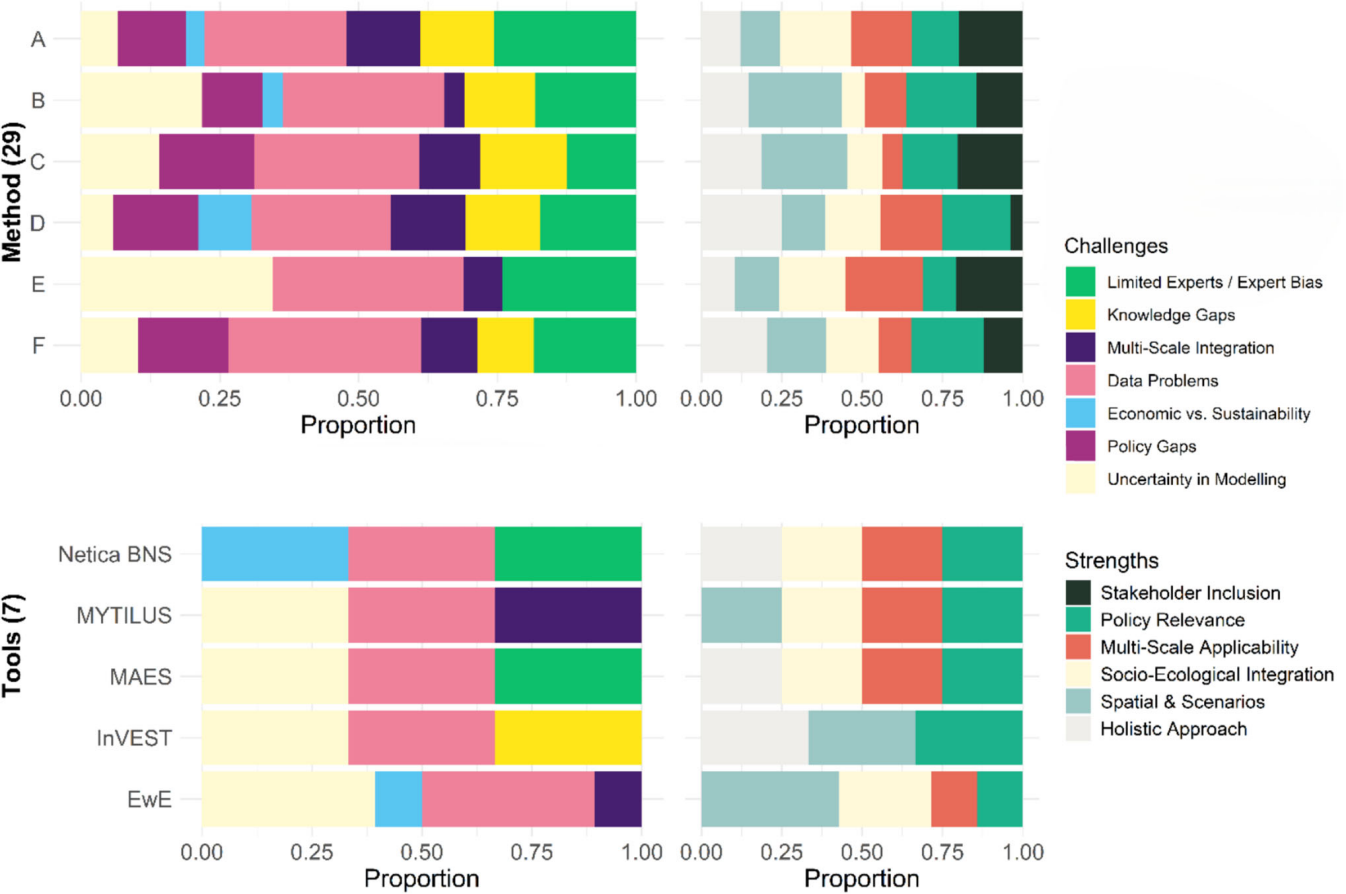
Challenges and strengths associated with different ESA methods and tools. Legend for the y-axis = **ESA Methods**: A. Systematic Review and Expert-Based Methodologies; B. Modeling-Based Approaches; C. Framework-Based Approaches; D. Economic Valuation and Decision-Support Approaches; E. Scenario-Based and Adaptive Management Approaches; F. Integrated and Holistic Approaches. **ESA Tools:** EwE. Ecopath with Ecosim; InVEST; MAES; MYTILUS; Netica BNS. Netica Bayesian Network software

The analysis of ESA methods reveals that each approach faces distinct challenges and offers specific strengths, reflecting the varied contexts and objectives in which they are applied within the reviewed studies (Fig. 6). In terms of challenges, all methods exhibit a similar distribution, indicating that the types of difficulties reported tend to recur regardless of the method applied in the cases reviewed. A particularly prominent challenge across all six methods is *Problems with Data—Gaps, Collection, and Reliability*, a category that appeared frequently in the reviewed sample and was common across geographical areas and activities (e.g., Veidemane et al. 2017).

It is also noteworthy that *Lack of Environmental Policy and Management Involvement* was not reported for the method category related to *Scenario-Based and Adaptive Management Approaches*. This absence could reflect the emphasis these case studies place on anticipating and incorporating policy aspects during design and application. Additionally, the challenge *Economic Interests vs. Sustainability Goals* was identified only in methods involving expert-based, modeling, and decision-support approaches (e.g., Maldonado et al. 2022).

In contrast, the strengths associated with each method varied depending on the socioeconomic and ecological contexts of the reviewed case studies. The strength related to *Spatial Analysis and Scenario Modeling* was particularly emphasized in methods that include this type of analytical component. For instance, long-run projections of integrated models were used to simulate the effects of alternative policy pathways on ecosystem pressures over time (Hyytiäinen et al. 2019), or to quantitatively assess management scenarios (Sundblad et al. 2020).

Regarding ESA tools (Fig. 6), a consistent challenge across all was *Problems with Data*—*Gaps, Collection, and Reliability*, as reported across most tool applications in the reviewed literature (Gasalla et al. 2004). The challenge related to *Lack of Environmental Policy and Management Involvement* was not commonly cited. Instead, this element was more frequently identified as a strength, which in the context of these case studies, may point to a stronger integration of policy considerations within tool-based applications.

### What is the effectiveness of different ESA methods and tools in supporting sustainable management?

The evaluation of the 35 case studies revealed notable variations in the effectiveness of ESA methods and tools, highlighting a range of strengths and limitations across case studies. Overall, 25 studies scored above 20 points, while 10 studies scored at or below this threshold (see SI—Annex F). The BS area recorded four cases below 20 points but also included the highest-scoring studies (e.g., von Thenen et al. 2023). Despite having fewer cases, the WMS registered only three studies below 20 points, with the top-scoring ones being Le Cornu et al. (2014) and Kincaid et al. (2017), both with 24 points. In the SAO case studies, the lowest values were observed, particularly in the case of Topor et al. (2019). However, one study (Firme Herbst et al. 2020) also achieved a score of 24 points, similar to the highest-scoring cases in the WMS. In the sample analyzed, cases reporting higher scores often corresponded to studies with more robust institutional involvement and greater access to datasets (Inácio et al. 2020), whereas lower scores tended to occur in cases where such conditions were not reported (Firme Herbst et al. 2020).

High-ranking studies, such as Kincaid et al. (2017), performed well by addressing all effectiveness principles, at least partially. Since all principles were weighted equally, studies that met multiple evaluation criteria (fully or partially) achieved higher scores. Case 31 (Armoskaitė et al. 2023) in the BS received the highest score (29 points) for comprehensively addressing all ten principles, fully meeting nine and partially addressing one. Its effectiveness stemmed from integrating multiple dimensions and scales, and a scalable, flexible approach.

Lower-ranking studies showed fewer positive evaluations in areas such as *Policy Relevance and Governance Support*, *Stakeholder Engagement and Inclusivity*, and *Practical Feasibility and Adaptability*. These aspects were less frequently addressed in certain case studies, which also reported challenges related to limited flexibility in data-deficient areas (e.g., Delfante de Padua Cardoso et al. 2020), or difficulty in addressing dynamic socio-ecological systems (e.g., Brun et al. 2024). In the reviewed cases, the diversity of methods and tools (along with the absence of standardized methodological frameworks) was associated with variability in results across areas and activities. As highlighted by Portman (2013), some studies have been designed to operate across spatial boundaries, which may support efforts to manage cumulative impacts in complex ecosystems.

Although some ESA methods demonstrated strengths in specific case study contexts, gaps were also evident in areas such as stakeholder engagement (Sundblad et al. 2020), practical feasibility (Ville d’Avray et al. 2019), and adaptability to changing conditions (Topor et al. 2019). These aspects were reported as limited in several studies, particularly in complex or interdisciplinary settings (e.g., Karydis 2023). In the context of the reviewed sample, aligning evaluation criteria more explicitly with core principles of effectiveness could enhance the capacity of ESA methods and tools to address diverse management contexts.

The effectiveness of ESA methods varied across marine activities, as assessed in the evaluation matrix presented in SI—Annex F. The fisheries-related studies generally exhibited the highest effectiveness scores (e.g., Inácio et al. 2020), reflecting strong integration of socioeconomic factors such as stakeholder interests and market conditions within ecological assessments (e.g., Ville d'Avray et al. 2019). MPA assessments showed a wider range of effectiveness, with some studies achieving high scores while others, particularly those from SAO (e.g., Topor et al. 2019), scored lower. ESA methods in MPAs case studies were especially effective in supporting planning efforts focused on conservation priorities and early components of the ES cascade, such as identifying ecosystem functions (e.g., Thrush and Dayton 2010). For the OWFs papers, ESA methods tended to yield intermediate effectiveness scores; they were often applied alongside other activities and were successful in assessing trade-offs between energy development and ecological impacts, although cumulative effects remained challenging to capture (e.g., Veidemane et al. 2017).

## Discussion

### Regional and activity-based variations in ESA applications

The analysis underscores the importance of contextual factors (both regional and sectoral) in shaping the application and effectiveness of ESA in marine environments. Differences across the studied regions reflect variation not only in ecological and socioeconomic conditions but also in institutional maturity, data availability, and the historical development of ESA practices (see Table 1). These contextual disparities condition both the feasibility and depth of ESA implementation. For instance, regions with higher institutional integration and better data infrastructure (such as the BS) tend to apply more advanced and model-based ESA approaches (e.g., Bastardie and Brown 2020). In contrast, data-limited regions, like parts of SAO, rely more on expert-based or descriptive assessments (e.g., Brun et al. 2024). These findings align with broader literature emphasizing that ESA implementation is contingent upon local capacities and governance settings (Uusitalo et al. 2023).

While the findings did not reveal strong regional contrasts in reported challenges or policy implications, this may stem from the limited and uneven distribution of case studies across regions. Expanding the geographical and sectoral coverage of ESA applications could further show how context mediates effectiveness. Nonetheless, the observed differences in ESA implementation support the argument that regional and activity-specific characteristics matter, and that effectiveness is not solely a function of methodological quality, but also of contextual fit.

Overall, the results suggest that improving ESA effectiveness requires approaches adapted to the governance realities, sectoral priorities, and data conditions of each context (Firme Herbst et al. 2020). This means refining methods for technical robustness while aligning them with decision-making frameworks and policy cycles (Brun et al. 2024). Strengthening capacities through sustained monitoring, collaborative networks, and open data is essential, alongside targeted investments in research infrastructure and long-term studies. As noted by Vieira Paiva et al. (2023), increased financial resources for research vessels, advanced technologies, and logistics are critical in data-limited regions, where funding gaps restrict study depth. Likewise, Topor et al. (2019) highlighted the need for expanded sampling to strengthen ESA’s ecological foundations.

One potential reason for differences in ESA design across activities is the degree to which they are well-established versus emerging. For instance, long-established uses such as fisheries (supported by historical data and regulatory frameworks) have enabled more integrated assessments that span the full ES cascade (Inácio et al. 2024). This contrasts with emerging sectors like OWFs case studies, where ESA applications remain limited and often focused on planning stages. Learning from sectors with longer ESA histories could help guide more robust assessments in newer marine uses. Sector-specific ESA applications thus allow for tailored evaluations, yet gaps persist (particularly in capturing trade-offs and synergies across coexisting marine activities) (Braat and de Groot 2012).

Building on these sectoral contrasts, the authors recommend that ESA methods be selected and further developed according to the maturity of the activity, the availability of data, and the nature of the trade-offs involved. In fisheries, where long-term datasets and regulatory frameworks exist, integrated approaches linking ecological values and socioeconomic outcomes have proven effective (Picone et al. 2017) and could guide the design of more robust ESA in data-limited sectors (Brun et al. 2024). The MPA case studies offer valuable insights into mapping beneficiaries and ecosystem service flows (Inácio et al. 2020) that could strengthen spatial planning in OWFs. Conversely, the socioeconomic emphasis in OWF assessments could enhance MPA assessments by better capturing multi-use interactions (Muñoz et al. 2018). Across all activities, remaining gaps include the integration of long-term monitoring, and the explicit evaluation of trade-offs and synergies between coexisting marine uses; elements essential for advancing ESA as a decision-support tool in marine governance (Oinonen et al. 2016).

Finally, the insights from the case studies echo longstanding challenges in operationalizing the ES cascade, which seeks to represent the complexity of ecological systems and their interactions with human well-being (Barbier et al. 2008). Despite progress, capturing nonlinear ecological dynamics and feedbacks remains methodologically demanding. While there is a clear call in the literature for more standardized ESA frameworks to enable comparability and consistency (Martín-López et al. 2014), the results presented here suggest that rigid standardization risks overlooking context-specific factors critical to effectiveness. ESA frameworks must therefore strike a balance, ensuring enough methodological consistency to support cross-case learning, while allowing flexibility to adapt to regional realities, an insight reinforced by this study’s comparative analysis across different marine sectors and governance contexts.

### Policy implications and governance considerations

The findings highlight critical insights for improving the policy relevance and practical utility of ESA in sustainable marine governance. For ESA to effectively inform decision-making, especially within MSP and sector-specific regulations, it must be tailored to the real-world priorities, institutional capacities, and governance in which it operates. While ESA frameworks have the potential to bridge ecological knowledge and management actions, their integration into policy remains limited, often due to a mismatch between methodological complexity and decision-making needs (Nahuelhual et al. 2020).

The limited presence of ESA in operational marine planning (such as the siting of OWF or the zoning of MPA) reveals a gap between assessment and implementation. ESA applications that explicitly account for policy trade-offs, spatial conflicts, and long-term sustainability goals are more likely to influence governance outcomes. For example, OWF-related ESA that incorporate spatial scenario modeling or cumulative impact assessments could directly support licensing processes and public engagement strategies (Inácio et al. 2020). Similarly, MPA design benefits from ESA methods that clarify ecosystem service flows and distributional effects, informing decisions on zoning and stakeholder inclusion (Topor et al. 2019).

To enhance ESA’s contribution to sustainable marine policy, assessments must be problem-oriented and embedded in planning cycles from the outset. In practice, strategic objectives vary across countries and do not always align with global or even national frameworks. For example, Firme Herbst et al. (2020) integrated subnational MSP processes and national governance strategies, strengthened by active stakeholder engagement at the regional level. Conversely, Vieira Paiva et al., (2023) illustrates that even when countries subscribe to the Sustainable Development Goals, activities may continue without effective control or adjustment to meet these targets. Aligning national objectives with global goals could provide benefits by enhancing policy coherence, facilitating access to international funding, and strengthening mechanisms for monitoring and reporting progress (Vieira Paiva et al. 2023). Achieving institutional commitment and policy receptiveness requires embedding ESA within established planning and coordination structures, fostering transparent stakeholder dialogue, and ensuring sustained investments in data systems to reduce uncertainty and support policy trade-offs.

Ultimately, improving the policy utility of ESA involves moving beyond descriptive assessments toward approaches that generate actionable knowledge. As Ressurreição et al. (2012) highlighted, scientists must communicate ecosystem service science in accessible formats that helps engage policymakers and the public by illustrating the ecological and economic consequences of both action and inaction. Effective ESA design should be informed by both scientific robustness and policy relevance, ensuring outputs support practical decision-making in marine systems characterized by complexity and change.

### Study transferability, methodological reflection, and future directions

This review offers a structured approach to assess how effectively ESA are implemented across contrasting marine governance contexts. Rather than aiming for exhaustive coverage, it focused on three regions and key marine activities to identify transferable patterns of strengths and limitations. The results highlight how contextual factors (such as data availability, institutional maturity, and sectoral priorities) shape ESA design, revealing recurring challenges (e.g., weak integration in planning) and practices that enhance effectiveness, including the early and active inclusion and engagement of stakeholders throughout the ESA process (Socrate et al. 2024).

The methodological evaluation applied here can be replicated in other marine contexts to evaluate assessment quality and applicability. Identifying cross-cutting trends (e.g., strengths in fisheries-related ESA, tool gaps in OWF) supports more targeted development of ESA, particularly for emerging sectors or underrepresented regions. To achieve this, applying the 10 principles of effectiveness can guide improvements, particularly by addressing gaps in stakeholder engagement (Sundblad et al. 2020), practical feasibility (Ville d’Avray et al. 2019), and adaptability to changing conditions (Topor et al. 2019). Incorporating these elements will help create more focused, context-appropriate, and effective ESA.

Complementing these methodological advances, the use of AI tools in systematic reviews can further enhance efficiency, consistency, and transparency, particularly when dealing with large volumes of literature. While human judgment remains essential, AI-assisted approaches offer promising avenues to support rigorous and reproducible assessment workflows.

Nevertheless, the limited number of case studies analyzed constrains the generalizability of the findings and highlights the need for broader regional and sectoral coverage in future research. Expanding the scope would strengthen the robustness of insights and contribute to refining typologies of ESA effectiveness. Additionally, incorporating new case studies across different regions could provide valuable understanding of how ESA practices evolve over time and within diverse governance contexts, shedding light on factors that influence policy uptake and the capacity for adaptation.

Ultimately, improving ESA implementation requires both context-specific adaptation and cross-site learning. Standardizing key assessment components (while allowing flexibility for local realities) can enhance comparability and inform sustainability efforts at different management scales. The evaluation methodology presented here offers practical value by guiding the selection of suitable ESA methods tailored to specific contexts and management objectives. For example, the evaluation table (SI—Annex F) enables targeted filtering of case studies by region, activity, and problems, helping practitioners identify relevant ESA approaches aligned with their goals. A case in point is the assessment of MPAs in the WMS region prioritizing scalability, flexibility, and interdisciplinary integration, where Cases 13 (Le Cornu et al. 2014) and 18 (Kincaid et al. 2017) emerge as strong candidates. While these cases demonstrate strengths in integration, their limitations in stakeholder engagement and adaptability underscore the evaluation methodology’s benefit in not only selecting appropriate methods but also highlighting critical areas for future improvement. Thus, this methodological pathway supports more strategic ESA design aligned with sustainable marine planning and policy needs.

## Conclusion

This study highlights how ESA supports sustainable marine management across different ecological and governance contexts. The analysis of the BS, WMS, and SAO revealed geographical variations driven by policy frameworks, data availability, and institutional capacities. While methodological diversity enhances ESA’s ability to capture complex socio-ecological interactions, inconsistencies in application and integration limit its effectiveness. Addressing these gaps is essential to strengthening ESA’s role in sustainable marine management.

Moving forward, enhancing ESA effectiveness will require refining methodological approaches, fostering interdisciplinary collaboration, and strengthening the link between scientific assessments and policy implementation. Ensuring that ESA remains a practical tool for decision-making depends on its ability to navigate trade-offs, integrate multiple knowledge systems, and adapt to region-specific governance needs. By providing insights into current ESA applications and their effectiveness, this study offers recommendations for future research and practical advancements in ecosystem-based marine planning.

## Supplementary Information

Below is the link to the electronic supplementary material.

Supplementary file1 (PDF 390 KB)

Supplementary file2 (XLSX 49 KB)

## Data Availability

The original contributions presented in this study are included in the article. Further inquiries can be directed to the corresponding author.

## References

[CR1] Alcamo, J., N. Ash, C. Butler, J. Callicott, D. Capistrano, S. Carpenter, J. Castilla, R. Chambers, et al. 2003. *Ecosystems and human well-being: A framework for assessment*. Island Press.

[CR2] Armoskaitė, A., J. Aigars, I. Andersone, I. M. Bonnevie, H. S. Hansen, S. Strake, M. von Thenen, L. Schrøder, et al. 2023. Setting the scene for a multi-map toolset supporting maritime spatial planning by mapping relative cumulative impacts on ecosystem service supply. *Frontiers in Marine Science* 10: 1–16. 10.3389/fmars.2023.1213119.

[CR3] Armoskaitė, A., I. Purina, J. Aigars, S. Strake, K. Pakalniete, P. Frederiksen, L. Schroder, and H. S. Hansen. 2020. Establishing the links between marine ecosystem components, functions and services: An ecosystem service assessment tool. *Ocean & Coastal Management* 193: 105229. 10.1016/j.ocecoaman.2020.105229.

[CR4] Barbier, E. B. 2017. Marine ecosystem services. *Current Biology* 27: R507–R510. 10.1016/j.cub.2017.03.020. 28586688 10.1016/j.cub.2017.03.020

[CR5] Barbier, E.B., E.W. Koch, B.R. Silliman, S.D. Hacker, E. Wolanski, J. Primavera, E. Granek, S. Polasky, et al. 2008. Coastal ecosystem-based management with nonlinear ecological functions and values. *Science*. 10.1126/science.1150349. 10.1126/science.1150349 18202288

[CR6] Bastardie, F., and E. J. Brown. 2020. Reverse the declining course: A risk assessment for marine and fisheries policy strategies in Europe from current knowledge synthesis. *Marine Policy* 126: 104409. 10.1016/j.marpol.2021.104409.

[CR7] Böhnke-Henrichs, A., C. Baulcomb, R. Koss, S. Salman Hussain, and R. de Groot. 2013. Typology and indicators of ecosystem services for marine spatial planning and management. *Journal of Environmental Management* 130: 135–145. 10.1016/j.jenvman.2013.08.027. 24076513 10.1016/j.jenvman.2013.08.027

[CR8] Braat, L., and R. de Groot. 2012. The ecosystem services agenda: Bridging the worlds of natural science and economics, conservation and development, and public and private policy. *Ecosystem Services* 1: 4–15. 10.1016/j.ecoser.2012.07.011.

[CR9] Brun, A., E. Verón, and J. Socrate. 2024. Land-sea-land interactions, with emphasis on fishing activity in the northern region of the Bonaerense Coastal Ecosystem, Argentina (In Spanish) (English summary). *Revista De Ciencias Ambientales* 58: 1–23. 10.15359/rca.58-2.3.

[CR10] Bryhn, A., P. Kraufvelin, U. Bergström, M. Vretborn, and L. Bergström. 2020. A model for disentangling dependencies and impacts among human activities and marine ecosystem services. *Environmental Management* 65: 575–586. 10.1007/s00267-020-01260-1. 32107570 10.1007/s00267-020-01260-1 PMC7145787

[CR11] Comisión Económica para América Latina y el Caribe (CEPAL). 2025. Panorama del océano, los mares y los recursos marinos y su contribución al desarrollo sostenible de América Latina y el Caribe (LC/TS.2025/30).

[CR12] Convention on Biological Diversity. 2022. Decision adopted by the Conference of the Parties to the Convention on Biological Diversity: CBD/COP/DEC/15/4. Kunming–Montreal. https://www.cbd.int/doc/decisions/cop-15/cop-15-dec-04-en.pdf.

[CR13] Corrales, X., D. Vilas, C. Piroddi, J. Steenbeek, J. Claudet, J. Lloret, A. Caló, A. Di Franco, et al. 2020. Multi-zone marine protected areas: Assessment of ecosystem and fisheries benefits using multiple ecosystem models. *Ocean & Coastal Management* 193: 105232. 10.1016/j.ocecoaman.2020.105232.

[CR14] Costanza, R., R. D’Arge, R. de Groot, S. Farber, M. Grasso, B. Hannon, K. Limburg, S. Naeem, et al. 1997. The value of the world’s ecosystem services and natural capital. *Nature* 387: 253–260. 10.1038/387253a0.

[CR15] de Groot, R. S., R. Alkemade, L. Braat, L. Hein, and L. Willemen. 2010. Challenges in integrating the concept of ecosystem services and values in landscape planning, management, and decision making. *Ecological Complexity* 7: 260–272. 10.1016/j.ecocom.2009.10.006.

[CR16] de Pádua, D., C. Cardoso, R. M. Formiga-Johnsson, R. Pinto de Lima, and R. de Oliveira Campos. 2020. Monitoring Human Activities in the Tamoios Ecological Station—Rio de Janeiro: Management Challenges. *Ambiente & Sociedade* 23: 1–22. 10.1590/1809-4422asoc20190112r2vu2020l5ao.

[CR17] Elliff, C., and R. K. P. Kukichi. 2017. Ecosystem services provided by coral reefs in a Southwestern Atlantic Archipelago. *Ocean & Coastal Management* 136: 49–55. 10.1016/j.ocecoaman.2016.11.021.

[CR18] Ervin, D., S. Vickerman, S. Ngawhika, F. Beaudoin, S. Hamlin, E. Dietrich, P. Manson, and J. Schoenen. 2014. Principles to Guide Assessments of Ecosystem Service Values first revised edition. Portland, Oregon: Cascadia Ecosystem Services Partnership, Institute for Sustainable Solutions, Portland State University.

[CR19] Everard, M. and R. Waters. 2013. Ecosystem services assessment: How to do one in practice (Version 1, October 13). Institution of Environmental Sciences, London. www.ies-uk.org.uk/resources/ecosystem-servicesassessment.

[CR20] Firme Herbst, D., L. Cavaleri Gerhardinger, D. Alves Vila-Nova, F. Grecco de Carvalho, and N. Hanazaki. 2020. Integrated and deliberative multidimensional assessment of a subtropical coastal-marine ecosystem (Babitonga bay, Brazil). *Ocean & Coastal Management* 196: 105279. 10.1016/j.ocecoaman.2020.105279.

[CR21] Firth, L. B., A. M. Knights, D. Bridger, A. J. Evans, N. Mieszkowska, P. J. Moore, N.E. O’Connor, E.V. Sheehan, et al. 2016. Ocean sprawl: Challenges and opportunities for biodiversity management in a changing world. In *Oceanography and Marine Biology: An Annual Review*, vol. 54, ed. R. N. Hughes, D. J. Hughes, I. P. Smith, and A. C. Dale. CRC Press.

[CR22] Flávio, H., R. Seitz, D. Eggleston, J. C. Svendsen, and J. Stottrup. 2023. Hard-bottom habitats support commercially important fish species: A systematic review for the North Atlantic Ocean and Baltic Sea. *PeerJ* 11: e14681. 10.7717/peerj.14681. 36684681 10.7717/peerj.14681 PMC9854379

[CR23] Gasalla, M. A., and C. L. D. B. Rossi-Wongtschwski. 2004. Contribution of ecosystem analysis to investigating the effects of changes in fishing strategies in the South Brazil Bight coastal ecosystem. *Ecological Modelling* 172: 283–306. 10.1016/j.ecolmodel.2003.09.012.

[CR24] Gee, K., N. Blazauskas, K. Dahl, C. Göke, B. Hassler, A. Kannen, N. Leposa, A. Morf, et al. 2019. Can tools contribute to integration in MSP? A comparative review of selected tools and approaches. *Ocean & Coastal Management* 179: 104834. 10.1016/j.ocecoaman.2019.104834.

[CR25] Grorud-Colvert, K., J. Sullivan-Stack, C. Roberts, V. Constant, B. Horta e Costa, E.P. Pike, N. Kingston, D. Laffoley, et al. 2021. The MPA Guide: A framework to achieve global goals for the ocean. *Science*. 10.1126/science.abf0861. 10.1126/science.abf0861 34516798

[CR26] Haddaway, N. R., M. J. Page, C. C. Pritchard, and L. A. McGuinness. 2022. PRISMA2020: An R package and Shiny app for producing PRISMA 2020-compliant flow diagrams, with interactivity for optimised digital transparency and Open Synthesis Campbell Systematic Reviews. *Campbell Systematic Reviews* 18: e1230. 10.1002/cl2.1230. 36911350 10.1002/cl2.1230 PMC8958186

[CR27] Hammer, M., C. M. Holmlund, and M. Aqvist Almlöv. 2003. Social-ecological feedback links for ecosystem management: A case study of fisheries in the Central Baltic Sea archipelago. *Ocean & Coastal Management* 46: 527–545. 10.1016/S0964-5691(03)00033-4.

[CR28] HELCOM. 2025. Annual Report 2024. Baltic Sea Environment Proceedings No. 201. HELCOM.

[CR29] Hyytiäinen, K., B. B. Bauer, K. Joyce, E. Ehrnsten, K. Eilola, B. G. Gustafsson, M. Meier, A. Norkko, et al. 2019. Provision of aquatic ecosystem services as a consequence of societal changes: The case of the Baltic Sea. *Population Ecology* 63: 61–74. 10.1002/1438-390X.12033.

[CR30] Inácio, M., L. Pinto, K. M. Baltranaitė, B. Burkhard, D. Barceló, and P. Pereira. 2024. Mapping and assessing marine ecosystem services supply in the Baltic Sea. *Science of the Total Environment* 950: 1–14. 10.1016/j.scitotenv.2024.175199. 10.1016/j.scitotenv.2024.175199 39102961

[CR31] Inácio, M., D. Karnauskaite, E. Baltranaité, M. Kalinauskas, K. Bogdzevic, E. Gomes, and P. Pereira. 2020. Ecosystem services of the Baltic Sea: An assessment and mapping perspective. *Geography and Sustainability* 1: 256–265. 10.1016/j.geosus.2020.11.001.

[CR32] Karydis, M. 2023. Toxic phytoplankton in eutrophic regional seas: An overview. *Global Nest Journal* 25: 178–211. 10.30955/gnj.005388.

[CR33] Kincaid, K., G. Rose, and R. Devillers. 2017. How fisher-influenced marine closed areas contribute to ecosystem-based management: A review and performance indicator scorecard. *Fish and Fisheries* 18: 860–876. 10.1111/faf.12211.

[CR34] Knight, A., R. Cowling, A. Boshoff, S. Wilson, and S. Pierce. 2011. Walking in STEP: Lessons for linking spatial prioritisations to implementation strategies. *Biological Conservation* 144: 202–211. 10.1016/j.biocon.2010.08.017.

[CR35] Koundouri, P., G. Halkos, C. F. M. Landis, and A. Alamanos. 2023. Ecosystem services valuation for supporting sustainable life below water. *Sustain Earth Reviews* 6: 1–6. 10.1186/s42055-023-00068-1. 10.1186/s42055-023-00068-1 PMC11728687 39807352

[CR36] Kukkala, A., and A. Moilanen. 2013. Core Concepts of spatial prioritization in systematic conservation planning. *Biological Reviews* 88: 434–464. 10.1111/brv.12008. 10.1111/brv.12008 PMC3654170 23279291

[CR37] Le Cornu, E., J. Kittinger, J. Zachary Koehn, E. M. Finkbeiner, and L. B. Crowder. 2014. Current Practice and Future Prospects for Social Data in Coastal and Ocean Planning. *Conservation Biology* 28: 902–911. 10.1111/cobi.12310. 24779578 10.1111/cobi.12310

[CR38] Lester, S. E., C. Costello, B. S. Halpern, S. D. Gaines, C. White, and J. A. Barth. 2013. Evaluation tradeoffs among ecosystem services to inform marine spatial planning. *Marine Policy* 38: 80–89. 10.1016/j.marpol.2012.05.022.

[CR39] Liquete, C., C. Piroddi, E. G. Drakou, L. Gurney, S. Katsanevakis, A. Charef, and B. Egoh. 2013. Current status and future prospects for the assessment of marine and coastal ecosystem services: A systematic review. *PLoS ONE* 8: 1–15. 10.1371/journal.pone.0067737. 10.1371/journal.pone.0067737 PMC3701056 23844080

[CR40] Maldonado, A. D., I. Galparsoro, G. Mandiola, I. de Santiago, R. Garnier, S. Pouso, A. Borja, I. Menchaca, et al. 2022. A Bayesian Network model to identify suitable areas for offshore wave energy farms, in the framework of ecosystem approach to marine spatial planning. *Science of the Total Environment* 838: 156037. 10.1016/j.scitotenv.2022.156037. 35598669 10.1016/j.scitotenv.2022.156037

[CR41] Martín-López, B., E. Gómez-Baggethun, M. García-Llorente, and C. Montes. 2014. Trade-offs across value-domains in ecosystem services assessment. *Ecological Indicators* 37: 220–228. 10.1016/j.ecolind.2013.03.003.

[CR42] Möllmann, C., and R. Diekmann. 2012. Marine Ecosystem Regime Shifts Induced by Climate and Overfishing: A Review for the Northern Hemisphere. *Advances in Ecological Research* 47: 303–347. 10.1016/B978-0-12-398315-2.00004-1.

[CR43] Muñoz, M., A. Reul, L. Gil de Sola, R. A. M. Lauerburg, O. Tello, A. Gimpel, and V. Stelzenmüller. 2018. A spatial risk approach towards integrated marine spatial planning: A case study on European hake nursery areas in the North Alboran Sea. *Marine Environmental Research* 142: 190–207. 10.1016/j.marenvres.2018.10.008. 30361105 10.1016/j.marenvres.2018.10.008

[CR44] Murphy, E. L., M. Bernard, L. R. Gerber, and K. J. Dooley. 2021. Evaluating the role of market-based instruments in protecting marine ecosystem services in wild-caught fisheries. *Ecosystem Services* 51: 101356. 10.1016/j.ecoser.2021.101356.

[CR45] Nahuelhual, L., X. Vergara, F. Bozzeda, C. Campos, M. D. Subida, L. Outeiro, S. Villasante, and M. Fernández. 2020. Exploring gaps in mapping marine ecosystem services: A benchmark analysis. *Ocean & Coastal Management* 192: 105193. 10.1016/j.ocecoaman.2020.105193.

[CR46] Oinonen, S., K. Hyytiäinen, L. Ahlvik, M. Laamanen, V. Lehtoranta, K. Salajärvi, and J. Virtanen. 2016. Cost-Effective Marine Protection—A Pragmatic Approach. *PLoS ONE* 11: 1–19. 10.1371/journal.pone.0147085. 10.1371/journal.pone.0147085 PMC4709167 26751965

[CR47] PICO Portal. 2024. Florida, United States. Available at www.picoportal.org.

[CR48] Picone, F., E. Buonocore, R. D’Agostaro, S. Donati, R. Chemello, and P. P. Franzese. 2017. Integrating natural capital assessment and marine spatial planning: A case study in the Mediterranean Sea. *Ecological Modelling* 361: 1–13. 10.1016/j.ecolmodel.2017.07.029.

[CR49] Pita, P., A.J. Castro, J.A. De Santiago-Meijide, M. Expósito-Granados, A. García-Allut, G. Méndez-Martínez, J. Molina-Urruela, J. Seijo, et al. 2024. Assessing the delivery of ecosystem services and benefits to human well-being of three contrasting MPAs in Spain. *Ecology and Society*. 10.5751/ES-15513-290419.

[CR50] Portman, M. E. 2013. Ecosystem services in practice: Challenges to real world implementation of ecosystem services across multiple landscapes – A critical review. *Applied Geography* 45: 185–192. 10.1016/j.apgeog.2013.09.011.

[CR51] Ressurreição, A., T. Zarzycki, M. Kaiser, G. Edwards-Jones, T. Ponce Dentinho, R. S. Santos, and J. Gibbons. 2012. Towards an ecosystem approach for understanding public values concerning marine biodiversity loss. *Marine Ecology Progress Series* 467: 15–28. 10.3354/meps09967.

[CR52] Scemama, P., C. Kermagoret, A. Accornero-Picon, F. Alban, P. Astruch, C. Boemare, C. F. Boudouresque, T. Changeux, et al. 2020. A Strategic Approach To Assess The Bundle Of Ecosystem Services Provided By Posidonia Oceanica Meadows In The Bay Of Marseille. *Vie Et Milieu-Life and Environment* 70: 197–207.

[CR53] Socrate, J., E. Verón, and G. García. 2024. Contributions to the planning of argentine maritime spaces: The Northern Patagonian socioecological system as a case study. *Marine Policy* 168: 106322. 10.1016/j.marpol.2024.106322.

[CR54] Steffen, W., K. Richardson, J. Rockström, S. E. Cornell, I. Fetzer, E. M. Bennett, R. Biggs, S.R. Carpenter, et al. 2015. Planetary boundaries: Guiding human development on a changing planet. *Science* 347: 736–747. 10.1126/science.1259855. 10.1126/science.1259855 25592418

[CR55] Sun, C., Y. Wang, and W. Zou. 2018. The marine ecosystem services values for China based on the emergy analysis method. *Ocean & Coastal Management* 161: 66–73. 10.1016/j.ocecoaman.2018.04.022.

[CR56] Sundblad, G., L. Bergström, T. Söderqvist, and U. Bergström. 2020. Predicting the effects of eutrophication mitigation on predatory fish biomass and the value of recreational fisheries. *Ambio* 49: 1090–1099. 10.1007/s13280-019-01263-1. 31598833 10.1007/s13280-019-01263-1 PMC7067735

[CR57] Thrush, S. F., and P. K. Dayton. 2010. What Can Ecology Contribute to Ecosystem-Based Management? *Annual Review of Marine Science* 2: 419–441. 10.1146/annurev-marine-120308-081129. 10.1146/annurev-marine-120308-081129 21141671

[CR58] Topor, Z. M., D. B. Rasher, J. E. Duffy, and S. J. Brandl. 2019. Marine protected areas enhance coral reef functioning by promoting fish biodiversity. *Conservation Letters* 12: 1–9. 10.1111/conl.12638.

[CR59] UNESCO-IOC. 2021. Technical Report on Current Conditions and Compatibility of Maritime Uses in the Western Mediterranean. Paris, UNESCO. (IOC Technical Series no 160).

[CR60] Uusitalo, L., R. Puntila-Dodd, J. Artell, and S. Jenberg. 2023. Modelling framework to evaluate societal effects of ecosystem management. *Science of the Total Environment* 898: 165508. 10.1016/j.scitotenv.2023.165508. 37442471 10.1016/j.scitotenv.2023.165508

[CR61] Veidemane, K., A. Ruskule, S. Strake, I. Purina, J. Aigars, S. Sprukta, D. Ustups, I. Putnis, et al. 2017. Application of the marine ecosystem services approach in the development of the maritime spatial plan of Latvia. *International Journal of Biodiversity Science Ecosystems Services & Management* 13: 398–411. 10.1080/21513732.2017.1398185.

[CR62] Vieira Paiva, S., P. Bastos Macedo Carneiro, T. Martins Garcia, T. C. Lopes Tavares, L. de Souza Pinheiro, A. Rodrigues Ximeenes Neto, T. C. Montalverne, et al. 2023. Marine carbonate mining in the Southwestern Atlantic: Current status, potential impacts, and conservation actions. *Marine Policy* 148: 105435. 10.1016/j.marpol.2022.105435.

[CR63] Ville d’Avray, L., D. Ami, A. Chenuil, R. David, and J. P. Feral. 2019. Application of the ecosystem service concept at a small-scale: The cases of coralligenous habitats in the North-western Mediterranean Sea. *Marine Pollution Bulletin* 138: 160–170. 10.1016/j.marpolbul.2018.10.057. 30660258 10.1016/j.marpolbul.2018.10.057

[CR64] von Thenen, M., N. Effelsberg, L. Weber, and G. Schrnewski. 2023. Perspectives and Scenarios for Coastal Fisheries in a Social-Ecological Context: An Ecosystem Service Assessment Approach in the German Baltic Sea. *Sustainability* 15: 1–19. 10.3390/su152215732.41704547

[CR65] White, C., B. S. Halpern, and C. V. Kappel. 2012. Ecosystem service tradeoff analysis reveals the value of marine spatial planning for multiple ocean uses. *PNAS* 109: 4696–4701. 10.1073/pnas.1114215109. 22392996 10.1073/pnas.1114215109 PMC3311352

[CR66] World Bank Group. 2025. Key Factors for Successful Development of Offshore Wind in Emerging Markets. ESMAP, World Bank, Washington, DC.

[CR67] Zaucha, J., K. Gee, E. Ramieri, L. Neimane, N. Alloncle, N. Blazauskas, H. Calado, C. Cervera-Núñez, et al. 2025. Implementing the EU MSP Directive: Current status and lessons learned in 22 EU Member States. *Marine Policy* 171: 1–23. 10.1016/j.marpol.2024.106425.

