# Supplementary material for: Towards improved ecosystem service assessments in marine systems: A systematic review and evaluation of effectiveness
Source: Ambio. 2025 Nov 7;55(7):1447–65. doi: 10.1007/s13280-025-02299-2 (PMC13230327; doi:10.1007/s13280-025-02299-2)
Supplement: Supplementary file 1 — Supplementary file1 (PDF 390 KB) [file 13280_2025_2299_MOESM1_ESM.pdf]

Ambio

Supplementary Information

Title: **Towards Improved Ecosystem Service Assessments in Marine Systems: A Systematic Review and Evaluation of Effectiveness**

Annex A

PRISMA Checklist applied to this review paper, based on Moher et al., 2010.

Section/topic	#	Checklist item	Reported on section #
**TITLE**			
Title	1	Identify the report as a systematic review, meta-analysis, or both.	Title
**ABSTRACT**			
Structured summary	2	Provide a structured summary including, as applicable: background; objectives; data sources; study eligibility criteria, participants, and interventions; study appraisal and synthesis methods; results; limitations; conclusions and implications of key findings; systematic review registration number.	Abstract
**INTRODUCTION**			
Rationale	3	Describe the rationale for the review in the context of what is already known.	1. Introduction
Objectives	4	Provide an explicit statement of questions being addressed with reference to participants, interventions, comparisons, outcomes, and study design (PICOS).	1. Introduction
**METHODS**			
Protocol and registration	5	Indicate if a review protocol exists, if and where it can be accessed (e.g., Web address), and, if available, provide registration information including registration number.	Section 2.2
Eligibility criteria	6	Specify study characteristics (e.g., PICOS, length of follow-up) and report characteristics (e.g., years considered, language, publication status) used as criteria for eligibility, giving rationale.	Section 2.2
Information sources	7	Describe all information sources (e.g., databases with dates of coverage, contact with study authors to identify additional studies) in the search and date last searched.	Section 2.2.1 and Annex B
Search	8	Present full electronic search strategy for at least one database, including any limits used, such that it could be repeated.	Annex B
Study selection	9	State the process for selecting studies (i.e., screening, eligibility, included in systematic review, and, if applicable, included in the meta-analysis).	Section 2.2.2
Data collection process	10	Describe method of data extraction from reports (e.g., piloted forms, independently, in duplicate) and any processes for obtaining and confirming data from investigators.	Section 2.3
Data items	11	List and define all variables for which data were sought (e.g., PICOS, funding sources) and any assumptions and simplifications made.	Annex D and E
Risk of bias in individual studies	12	Describe methods used for assessing risk of bias of individual studies (including specification of whether this was done at the study or outcome level), and how this information is to be used in any data synthesis.	Section 2.3 and 2.4
Summary measures	13	State the principal summary measures (e.g., risk ratio, difference in means).	Not applicable
Synthesis of results	14	Describe the methods of handling data and combining results of studies, if done, including measures of consistency (e.g., I^2) for each meta-analysis.	Section 2.3.2
Risk of bias across studies	15	Specify any assessment of risk of bias that may affect the cumulative evidence (e.g., publication bias, selective reporting within studies).	Section 2.3 and 2.4
Additional analyses	16	Describe methods of additional analyses (e.g., sensitivity or subgroup analyses, meta-regression), if done, indicating which were pre-specified.	Section 2.3.2 and 2.4

**RESULTS**			
Study selection	17	Give numbers of studies screened, assessed for eligibility, and included in the review, with reasons for exclusions at each stage, ideally with a flow diagram.	Section 2.2 and Annex C
Study characteristics	18	For each study, present characteristics for which data were extracted (e.g., study size, PICOS, follow-up period) and provide the citations.	Section 3 and Annex C
Risk of bias within studies	19	Present data on risk of bias of each study and, if available, any outcome level assessment (see item 12).	Not applicable
Results of individual studies	20	For all outcomes considered (benefits or harms), present, for each study: (a) simple summary data for each intervention group (b) effect estimates and confidence intervals, ideally with a forest plot.	Not applicable
Synthesis of results	21	Present the main results of the review. If meta-analyses are done, include for each, confidence intervals and measures of consistency.	Section 3
Risk of bias across studies	22	Present results of any assessment of risk of bias across studies (see Item 15).	Not applicable
Additional analysis	23	Give results of additional analyses, if done (e.g., sensitivity or subgroup analyses, meta-regression [see Item 16]).	Section 3
**DISCUSSION**			
Summary of evidence	24	Summarize the main findings including the strength of evidence for each main outcome; consider their relevance to key groups (e.g., healthcare providers, users, and policy makers).	Section 4
Limitations	25	Discuss limitations at study and outcome level (e.g., risk of bias), and at review-level (e.g., incomplete retrieval of identified research, reporting bias).	Section 4
Conclusions	26	Provide a general interpretation of the results in the context of other evidence, and implications for future research.	Section 4
**FUNDING**			
Funding	27	Describe sources of funding for the systematic review and other support (e.g., supply of data); role of funders for the systematic review.	Title page – Funding

Reference:

Moher D, Liberati A, Tetzlaff J, Altman DG (2010) Preferred reporting items for systematic reviews and meta-analyses: the PRISMA statement. International journal of surgery (London, England) 8: 336–341.

Annex B

Searcher, Search String, and Number of Results. The search was performed on 06.08.2024.

Searcher	String	No. Of results
Web of Science	((TI=("Ecosystem service assessment*" OR "ESA method*" OR "ecosystem-based approach*" OR "ecosystem-based management*" OR "ecosystem services evaluation*" OR "ecosystem services assessment*" OR "Ecosystem approach*") OR AB=("Ecosystem service assessment*" OR "ecosystem-based approach*" OR "ecosystem-based management*" OR "ecosystem services evaluation*" OR "ecosystem services assessment*" OR "Ecosystem approach*") OR WC=("Ecosystem service assessment*" OR "ESA method*" OR "ecosystem-based approach*" OR "ecosystem-based management*" OR "ecosystem services evaluation*" OR "ecosystem services assessment*" OR "Ecosystem approach*")))	**3582**
	OR ((TI=("ecosystem service*" OR "ecosystem function*" OR "ecosystem benefit*" OR "ecological service*") OR AB=("ecosystem service*" OR "ecosystem function*" OR "ecosystem benefit*" OR "ecological service*") OR WC=("ecosystem service*" OR "ecosystem function*" OR "ecosystem benefit*" OR "ecological service*")))	
	AND ((TI=("Baltic Sea*" OR "South Atlantic Ocean*" OR "Southern Atlantic Ocean*" OR "Southwestern Atlantic Ocean*" OR "SW Atlantic Ocean*" OR "South America Atlantic Coast*" OR "South America Marine*" OR "Western Mediterranean Sea*" OR "Western Med*") OR AB=("Baltic Sea*" OR "South Atlantic Ocean*" OR "Southern Atlantic Ocean*" OR "Southwestern Atlantic Ocean*" OR "SW Atlantic Ocean*" OR "South America Atlantic Coast*" OR "South America Marine*" OR "Western Mediterranean Sea*" OR "Western Med*") OR WC=("Baltic Sea*" OR "South Atlantic Ocean*" OR "Southern Atlantic Ocean*" OR "Southwestern Atlantic Ocean*" OR "SW Atlantic Ocean*" OR "South America Atlantic Coast*" OR "South America Marine*" OR "Western Mediterranean Sea*" OR "Western Med*")))	

	AND ((TI=("fishing*" OR "fishery*" OR "marine protected areas*" OR "aquaculture*" OR "fish farms*" OR "offshore wind farms*" OR "marine renewable energy*") OR AB=("fishing*" OR "fishery*" OR "marine protected areas*" OR "aquaculture*" OR "fish farms*" OR "offshore wind farms*" OR "marine renewable energy*") OR WC=("fishing*" OR "fishery*" OR "marine protected areas*" OR "aquaculture*" OR "fish farms*" OR "offshore wind farms*" OR "marine renewable energy*")))	
SCOPUS	(TITLE-ABS-KEY ("Ecosystem service assessment*" OR "ecosystem-based approach*" OR "ecosystem-based management*" OR "ecosystem services evaluation*" OR "ecosystem services assessment*" OR "Ecosystem approach*")) OR (TITLE-ABS-KEY ("ecosystem service*" OR "ecosystem function*" OR "ecosystem benefit*" OR "ecological service*")) AND (TITLE-ABS-KEY ("Baltic Sea*" OR "South Atlantic Ocean*" OR "Southern Atlantic Ocean*" OR "Southwestern Atlantic Ocean*" OR "SW Atlantic Ocean*" OR "South America Atlantic Coast*" OR "Western Mediterranean Sea*" OR "Western Med*")) AND (TITLE-ABS-KEY ("fishing*" OR "fishery*" OR "marine protected areas*" OR "aquaculture*" OR "fish farms*" OR "offshore wind farms*" OR "marine renewable energy*"))	**300**
SCIELO	(("Evaluación de servicios ecosistémicos*" OR "enfoque basado en ecosistemas*" OR "gestión basada en ecosistemas*")) OR ((servicio ecosistémico) OR (servicio ecológico)) OR ((pesca) OR (pesquería) OR (áreas marinas protegidas) OR (granjas eólicas marinas) OR (energía renovable marina)) AND (BRASIL)	**116**
	(("Evaluación de servicios ecosistémicos*" OR "enfoque basado en ecosistemas*" OR "gestión basada en ecosistemas*")) OR ((servicio ecosistémico) OR (servicio ecológico)) OR ((pesca) OR (pesquería) OR (áreas marinas protegidas) OR (granjas eólicas marinas) OR (energía renovable marina)) AND (ARGENTINA)	**78**
	(("Evaluación de servicios ecosistémicos*" OR "enfoque basado en ecosistemas*" OR "gestión basada en ecosistemas*")) OR ((servicio ecosistémico) OR (servicio ecológico)) OR ((pesca) OR (pesquería) OR (áreas marinas protegidas) OR (granjas eólicas marinas) OR (energía renovable marina)) AND (URUGUAY)	**2**

Annex C

Bibliographic references of the 35 case studies included in the review

1. Armoskaité, A., Aigars, J., Andersone, I., Bonnevie, I. M., Hansen, H. S., Strake, S., von Thenen, M., Schroder, L. (2023). Setting the scene for a multi-map toolset supporting maritime spatial planning by mapping relative cumulative impacts on ecosystem service supply. Frontiers in Marine Science, 10. https://doi.org/10.3389/fmars.2023.1213119
2. Armoskaité, A., Purina, I., Aigars, J., Strake, S., Pakalniete, K., Frederiksen, P., Schroder, L., Hansen, H. S. (2020). Establishing the links between marine ecosystem components, functions and services: An ecosystem service assessment tool. Ocean & Coastal Management, 193. https://doi.org/10.1016/j.ocecoaman.2020.105229
3. Bastardie, F. & Brown, E. J. (2021). Reverse the declining course: A risk assessment for marine and fisheries policy strategies in Europe from current knowledge synthesis. Marine Policy, 126. https://doi.org/10.1016/j.marpol.2021.104409
4. Brun, A., Verón, E., Socrate, J. (2024). Interacciones tierra-mar-tierra, con énfasis en la actividad pesquera en la región norte del Ecosistema Costero Bonaerense, Argentina. Revista de Ciencias Ambientales, 58(2). https://doi.org/10.15359/rca.58-2.3
5. Bryhn, A., Kraufvelin, P., Bergström, U., Vretborn, M., Bergström, L. (2020). A Model for Disentangling Dependencies and Impacts among Human Activities and Marine Ecosystem Services. ENVIRONMENTAL MANAGEMENT, 65(5). https://doi.org/10.1007/s00267-020-01260-1
6. Corrales, X., Vilas, D., Piroddi, C., Steenbeek, J., Claudet, J., Lloret, J., Caló, A., Di Franco, A., Font, T., Ligas, A., Prato, G., Sahyoun, R., Sartor, P., Guidetti, P., Coll, M. (2020). Multi-zone marine protected areas: Assessment of ecosystem and fisheries benefits using multiple ecosystem models. Ocean & Coastal Management, 193. https://doi.org/10.1016/j.ocecoaman.2020.105232
7. Delfante de Pádua Cardoso, C., Formiga-Johnsson, R. M., Pinto de Lima, R., de Oliveira Campos, R. (2020). Monitoring Human Activities in the Tamoios Ecological Station—Rio de Janeiro: Management Challenges. Ambiente & Sociedade, 23. https://doi.org/10.1590/1809-4422asoc20190112r2vu2020l5ao
8. Elliff, C., Kukichi, R. K. P. (2017). Ecosystem services provided by coral reefs in a Southwestern Atlantic Archipelago. OCEAN \& COASTAL MANAGEMENT, 136. https://doi.org/10.1016/j.ocecoaman.2016.11.021
9. Firme Herbst, D., Cavaleri Gerhardinger, L., Alves Vila-Nova, D., Grecco de Carvalho, F., Hanazaki, N. (2020). Integrated and deliberative multidimensional assessment of a subtropical coastal-marine ecosystem (Babitonga bay, Brazil). OCEAN \& COASTAL MANAGEMENT, 196. https://doi.org/10.1016/j.ocecoaman.2020.105279
10. Firth, L. B., Knights, A. M., Bridger, D., Evans, A. J., Mieszkowska, N., Moore, P. J., O'Connor, N. E., Sheehan, E. V., Thompson, R. C., Hawkins, S. J. (2016). OCEAN SPRAWL: CHALLENGES AND OPPORTUNITIES FOR BIODIVERSITY MANAGEMENT IN A CHANGING WORLD. 54, 193–269.
11. Flávio, H., Seitz, R., Eggleston, D., Svendsen, J. C., Stottrup, J. (2023). Hard-bottom habitats support commercially important fish species: A systematic review for the North Atlantic Ocean and Baltic Sea. PeerJ, 11. https://doi.org/10.7717/peerj.14681
12. Gasalla, M. A., Rossi-Wongtschwski, C. L. D. B. (2004). Contribution of ecosystem analysis to investigating the effects of changes in fishing strategies in the South Brazil Bight coastal ecosystem. ECOLOGICAL MODELLING, 172(2). https://doi.org/10.1016/j.ecolmodel.2003.09.012
13. Hammer, M., Holmlund, C. M., Aqvist Almlöv, M. (2003). Social-ecological feedback links for ecosystem management: A case study of fisheries in the Central Baltic sea archipelago. Ocean and Coastal Management, 46(6). https://doi.org/10.1016/S0964-5691(03)00033-4
14. Hyytiäinen, K., Bauer, B. Bly Joyce, K., Ehrnsten, E., Eilola, K., Gustafsson, B. G., Meier, M., Norkko, A., Saraiva, S., Tomczak, M., Zandersen, M. (2021). Provision of aquatic ecosystem services as a consequence of societal changes: The case of the Baltic Sea. Population Ecology, 63(1). https://doi.org/10.1002/1438-390X.12033
15. Inácio, M., Karnauskaite, D., Baltranaité, E., Kalinauskas, M., Bogdzevic, K., Gomes, E., Pereira, P. (2020). Ecosystem services of the Baltic Sea: An assessment and mapping perspective. Geography and Sustainability, 1(4). https://doi.org/10.1016/j.geosus.2020.11.001
16. Karydis, M. (2023). Toxic phytoplankton in eutrophic regional seas: An overview. Global Nest Journal, 25(1), 178. https://doi.org/10.30955/gnj.005388
17. Kincaid, K., Rose, G., Devillers, R. (2017). How fisher-influenced marine closed areas contribute to ecosystem-based management: A review and performance indicator scorecard. FISH AND FISHERIES, 18(5). https://doi.org/10.1111/faf.12211
18. Le Cornu, E., Kittinger, J., Zachary Koehn, J., Finkbeiner, E. M., Crowder, L. B. (2014). Current Practice and Future Prospects for Social Data in Coastal and Ocean Planning. CONSERVATION BIOLOGY, 28(4). https://doi.org/10.1111/cobi.12310

19. Maldonado, A. D., Galparsoro, I., Mandiola, G., de Santiago, I., Garnier, R., Pouso, S., Borja, A., Menchaca, I., Marina, D., Zubiate, L., Bald, J. (2022). A Bayesian Network model to identify suitable areas for offshore wave energy farms, in the framework of ecosystem approach to marine spatial planning. SCIENCE OF THE TOTAL ENVIRONMENT, 838(2). https://doi.org/10.1016/j.scitotenv.2022.156037
20. Möllmann, C. Diekmann, R. (2012). Marine Ecosystem Regime Shifts Induced by Climate and Overfishing: A Review for the Northern Hemisphere. 47. https://doi.org/10.1016/B978-0-12-398315-2.00004-1
21. Muñoz, M., Reul, A., Gil de Sola, L., Lauerburg, R. A. M., Tello, O., Gimpel, A., Stelzenmüller, V. (2018). A spatial risk approach towards integrated marine spatial planning: A case study on European hake nursery areas in the North Alboran Sea. Marine Environmental Research, 142. PubMed. https://doi.org/10.1016/j.marenvres.2018.10.008
22. Oinonen, S., Hyytiäinen, K., Ahlvik, L., Laamanen, M., Lehtoranta, V. Salajärvi, K., Virtanen, J. (2016). Cost-Effective Marine Protection—A Pragmatic Approach. PLOS ONE, 11(1). https://doi.org/10.1371/journal.pone.0147085
23. Picone, F., Buonocore, E., D'Agostaro, R., Donati, S., Chemello, R., Franzese, P. P.(2017). Integrating natural capital assessment and marine spatial planning: A case study in the Mediterranean sea. Ecological Modelling, 361. https://doi.org/10.1016/j.ecolmodel.2017.07.029
24. Ressurreicao, A., Zarzycki, T., Kaiser, M., Edwards-Jones, G., Ponce Dentinho, T., Santos, R. S., Gibbons, J. (2012). Towards an ecosystem approach for understanding public values concerning marine biodiversity loss. MARINE ECOLOGY PROGRESS SERIES, 467, 15. https://doi.org/10.3354/meps09967
25. Scemama, P., Kermagoret, C., Accornero-Picon, A., Alban, F., Astruch, P., Boemare, C., Boudouresque, C. F., Changeux, T., Charbonnel, E., Harmelin-Vivien, M., Le Direach, L., Mongruel, R., Ourgaud, M., Ruitton, S., Verlaque, M. (2020). A STRATEGIC APPROACH TO ASSESS THE BUNDLE OF ECOSYSTEM SERVICES PROVIDED BY POSIDONIA OCEANICA MEADOWS IN THE BAY OF MARSEILLE. VIE ET MILIEU-LIFE AND ENVIRONMENT, 70(3), 197–207.
26. Socrate, J., Veron, E. (2022) Analysis of uses and activities in the Argentine Sea. Bases for a Marine Spatial Planning in the North Argentina Basin. Mar. Policy 139 https://doi.org/10.1016/j.marpol.2022.105014.
27. Socrate, J., Verón, E., García, G. (2024). Contributions to the planning of argentine maritime spaces: The Northern Patagonian socioecological system as a case study. Marine Policy, 168. https://doi.org/10.1016/j.marpol.2024.106322
28. Sundblad, G., Bergström, L., Söderqvist, T., Bergström, U. (2020). Predicting the effects of eutrophication mitigation on predatory fish biomass and the value of recreational fisheries. Ambio, 49(5), 1090. https://doi.org/10.1007/s13280-019-01263-1
29. Thrush, S. F., Dayton, P. K. (2010). What Can Ecology Contribute to Ecosystem-Based Management? ANNUAL REVIEW OF MARINE SCIENCE, 2, 419–441. https://doi.org/10.1146/annurev-marine-120308-081129
30. Topor, Z. M., Rasher, D. B., Duffy, J. E., Brandl, S. J. (2019). Marine protected areas enhance coral reef functioning by promoting fish biodiversity. CONSERVATION LETTERS, 12(4). https://doi.org/10.1111/conl.12638
31. Uusitalo, l., Puntila-Dodd, R., Artell, J., Jenberg, S. (2023). Modelling framework to evaluate societal effects of ecosystem management. Science of the Total Environment, 898. https://doi.org/10.1016/j.scitotenv.2023.165508
32. Veidemane, K., Ruskule, A., Strake, S., Purina, I., Aigars, J., Sprukta, S., Ustups, D., Putnis, I., Klepers, A. (2017). Application of the marine ecosystem services approach in the development of the maritime spatial plan of Latvia. International Journal of Biodiversity Science Ecosystems Services & Management, 13(1). https://doi.org/10.1080/21513732.2017.1398185
33. Vieira Paiva, S:, Bastos Macedo Carneiro, P., Martins Garcia, T., Lopes Tavares, T. C., de Souza Pinheiro, L., Rodrigues Ximeenes Neto, A., Montalverne, T. C., Soares, M. O. (2023). Marine carbonate mining in the Southwestern Atlantic: Current status, potential impacts, and conservation actions. Marine Policy, 148. https://doi.org/10.1016/j.marpol.2022.105435
34. Ville d'Avray, L., Ami, d., Chenuil, A., David, R., Feral, J. P. (2019). Application of the ecosystem service concept at a small-scale: The cases of coralligenous habitats in the North-western Mediterranean Sea. Marine Pollution Bulletin, 138, 160. https://doi.org/10.1016/j.marpolbul.2018.10.057
35. von Thenen, M., Effelsberg, N., Weber, L., Schrnewski, G. (2023). Perspectives and Scenarios for Coastal Fisheries in a Social-Ecological Context: An Ecosystem Service Assessment Approach in the German Baltic Sea. Sustainability (Switzerland), 15(2). https://doi.org/10.3390/su152215732

Annex D

Criteria for Data Extraction Process, uploaded as metadata to Pico Portal software, for the data extraction process.

General Information

- **Cite ID**: Number assigned to the paper by PICO software for referencing purposes.
- **Article Title**: Title of the research article.
- **Year of Publication**: The year in which the article was published.
- **General Aim**: The primary objective or purpose of the research, mentioned in the abstract or in the main text.
- **Cite**: Formal reference to the article, formatted according to APA rules.

Importance for objectives of Review

- **Geographical Region**: The three main general groups studied: Baltic Sea, Western Mediterranean Sea, South Atlantic Ocean.
- **Specific Geographical Region**: The specific location cited in the paper (e.g., German Baltic Sea, Alboran Sea, Argentinian Sea).
- **Marine activity**: The main activities mentioned in the study, such as fishing, offshore wind farms (OWF), and/or Marine Protected Areas (MPA).
- **Specific Marine Activity (MA)**: Any other activities addressed in the paper (if applicable), which may not be present if the focus is solely on one or more of the main activities.
- **ESA Method**: A method in ESA represents the overarching systematic approach or framework used to evaluate ecosystem services. It involves a series of structured steps and procedures that guide how to conduct the assessment. Methods provide the foundational strategy, defining the comprehensive sequence of activities necessary for gathering, processing, and analyzing data (this may not be present if the paper discusses only a tool).
- **ESA Tool**: A tool is a specific instrument or application used within a method to perform particular functions in the evaluation process. Tools are tangible resources such as software, databases, models, or techniques that operationalize the method's procedures (this may not be present if the paper discusses only a method).
- **Ecosystem services Classification used**: The classification considered for categorizing ecosystem services, such as Millennium Ecosystem Assessment (MA), Common International Classification of Ecosystem Services (CICES), The Economics of Ecosystems and Biodiversity (TEEB), etc.

Result information

- **Type of habitat analyzed**: The type of ecosystem analyzed in the study, such as coastal, marine, or coastal-marine.
- **Ecosystem Services (ES) Analyzed**: The number and types of ecosystem services that were analyzed in the research.
- **Mention of Ecosystem Services (ES) Cascade? If so, how**: Indicates whether the analysis considers the application of the ecosystem services cascade (Haines-Young and Potschin, 2010). If yes, it should detail how the methodology is utilized in the analysis.
- **Which Part of the ES Cascade is the Paper Involved?:** Whether or not the cascade is mentioned, and considering the following elements and definitions of the cascade, identify in which stage(s) the paper's analysis can be categorized: ecosystem components or ecosystem structures, ecosystem functions and processes, ecosystem services, values, and benefits (Haines-Young and Potschin, 2010; Liquete et al, 2013)
- **Mention of Trade-offs?**: Indicates whether trade-offs are discussed in the paper: how they are defined, what is understood by them, and what results are presented.
- **Policy Implications**: How the results of the paper relate to the application of policies (current or future/suggested by the authors).

- **Strengths or Benefits of the Methodology Application**: The advantages brought by applying the ESA method or tool used in the study.
- **Challenges/Limitations of the Methodology Application**: The challenges or limitations encountered when applying the methodology.

Methodological information

- **General Conceptual Framing**: The main concepts that guide the theoretical framework of the research.
- **Methodology**: The specific methodology applied in the paper, outlining the structured approach taken to conduct the research.
- **Qualitative, Quantitative, or Mixed Analyses**: Specifies whether the analyses conducted are qualitative, quantitative, or a combination of both.
- **Time Scale**: Describes the temporal aspect of the study, which may include historical changes, a single point in time, short and medium-term future scenarios, and long-term futures (Dang et al., 2021).
- **Spatial Scale of the Case Study**: The geographical scope of the study, classified as local, subnational, national, supranational, continental, or global (Liquete et al., 2013).
- **Integration of Ecosystem Services (ES) Concept**: This refers to how the ES concept is utilized in the paper, whether as an analytical category or merely as a general concept framing the research.
- **Type of Data**: Specifies the types of data utilized in the research, which may include primary data, simple statistics, model data, proxy data, or expert opinions (Liquete et al., 2013).

Other

- **Notes**: Any information that is relevant but specific to each paper that does not fit into the previously analyzed categories. This may include unique observations, additional insights, or contextual information that adds value to the understanding of the research but is not categorized elsewhere.

Annex G

Relationships between key problems, specific evaluation questions, guiding principles, and which of the 23 PICO-extracted categories was used to assess the effectiveness of ESA methods and tools in the reviewed case studies.

Problems	Specific Questions	Principles of Effectiveness	Category to answer it
Problem 1: Multi-Scale and Multi-Dimensional Integration	1.1 Does the method/tool address multiple geopolitical and/or geographical scales in its analysis?	Scalability and Flexibility	Spatial Scale
	1.2 Is the method/tool adaptable to different scales (local, regional, global)?	Scalability and Flexibility	Spatial scale<br>Strengths
	1.3 Does it integrate ecological, social, and economic dimensions effectively?	Integration of Multiple Dimensions	General Conceptual Framing<br>Integration of ES Concept<br>Strengths
Problem 2: Policy Relevance and Governance Support	2.1 Does the method/tool produce results directly relevant to policy-making?	Policy Relevance	General Aim<br>Strengths<br>Policy implications
	2.2 Does it help in aligning with national and/or transnational policies?	Policy Relevance	Policy implications<br>Geographical Region
	2.3 Does it offer actionable insights for spatial and temporal management (e.g., MSP)?	Policy Relevance	General Aim<br>Time Scale<br>Spatial Scale
Problem 3: Stakeholder Engagement and Inclusivity	3.1 Does the method/tool explicitly include stakeholder engagement in the assessment process?	Stakeholder Engagement	Strengths<br>Policy implications
	3.2 Does it incorporate marginalized or less-represented stakeholder groups?	Stakeholder Engagement	Strengths
	3.3 Does it enhance perceived legitimacy of decisions among stakeholders?	Stakeholder Engagement	Policy implications
Problem 4: Knowledge Integration and Scientific Robustness	4.1 Does the method/tool address specific knowledge gaps in ESA?	Comprehensive Scope	Challenges
	4.2 Is the methodology scientifically rigorous, based on empirical data?	Scientific Rigor	Type of data<br>Challenges
	4.3 Does it provide transparent and reproducible processes?	Scientific Rigor<br>Transparency and Reproducibility	Methodology type<br>General Conceptual Framing
Problem 5: Practical Feasibility and Adaptability	5.1 Is the method/tool practical in terms of data, time, and expertise required?	Practical Feasibility	Type of data<br>Challenges
	5.2 Can it adapt to new data or changing ecosystem conditions over time?	Dynamic and Adaptive	Class Method/Tool<br>Policy implications
	5.3 Does it assess future scenarios or long-term sustainability?	Dynamic and Adaptive	Class Method/Tool<br>Challenges

Problem 6: Comprehensive Scope and Ecosystem Approach	6.1 Does the method/tool assess the full range of ES (provisioning, regulating, cultural, and supporting)?	Comprehensive Scope	Ecosystem Services Classification<br>ES Cascade Part
	6.2 Does it employ a holistic, interdisciplinary approach in its evaluation?	Integration of Multiple Dimensions<br>Interdisciplinary Approach	Strengths<br>Class Method/Tool
	6.3 Does it reflect an ecosystem-based management perspective?	Comprehensive Scope	General Aim<br>Strengths
